# Using electronic health records to understand multimorbidity in older people: a scoping review

**DOI:** 10.1007/s41999-025-01231-x

**Published:** 2025-07-03

**Authors:** Lucy Smith, Glenn Simpson, Neil Singh, Lucy Wells, Hajira Dambha-Miller

**Affiliations:** University of Southampton, Southampton, UK

**Keywords:** EHR, Multimorbidity, Older people, Scoping review

## Abstract

**Purpose:**

The increasing availability of electronic health records (EHR), encompassing routinely collected general practice, hospital, and linked national census data, presents significant opportunities to enhance our understanding of multimorbidity at scale. However, the utility and clinical impact of EHR in advancing multimorbidity research, particularly concerning older adults who are disproportionately affected, remain unclear. This study aims to synthesise the literature on the use of EHR to investigate multimorbidity in individuals aged 65 years and older.

**Method:**

We conducted a scoping review, performing literature searches across Medline, Embase, CINAHL, and the Cochrane Central Register of Controlled Trials. Studies published from the inception of these databases until September 2024 were included if they investigated multimorbidity using EHR in individuals aged 65 years and older. The extracted data were synthesised using an iterative descriptive process.

**Results:**

A total of 753 studies were identified, with 46 meeting the eligibility criteria for inclusion. All studies originated from high-income countries, primarily the USA and Spain. Research focused on areas such as healthcare utilisation and costs, predictive modelling based on patient disease profiles, specific clustering models, relationships between multimorbidity and particular disease categories, aetiology, polypharmacy, and the accuracy of EHR. Reporting on data completeness, sociodemographic characteristics, and the inclusion of ethnic and social diversity within EHR datasets was notably limited.

**Conclusions:**

The use of EHR to elucidate multimorbidity is expanding and holds substantial promise. However, enhanced reporting of data completeness and sociodemographic characteristics is essential to facilitate the effective translation of findings into real-world populations. Neglecting these aspects, risks exacerbating inequality gaps for individuals 65 years and older with multimorbidity.

**Supplementary Information:**

The online version contains supplementary material available at 10.1007/s41999-025-01231-x.

## Key summary points

**Aim:**

This study aims to synthesise the existing literature on the utility of electronic health records (EHR) to understand multimorbidity among older adults aged 65 years and above.

**Findings:**

EHR are increasingly utilised to investigate multimorbidity. However, the current body of research predominantly focuses on limited populations within high-income countries. There is a notable scarcity of studies reporting comprehensive sociodemographic characteristics, with few addressing the socioeconomic and ethnic diversity essential for a nuanced understanding of multimorbidity.

**Message:**

While EHR demonstrates considerable potential in identifying high-risk groups and patterns of multimorbidity, there is a risk of exacerbating health disparities. The limitations of EHR systems in adequately capturing data from diverse populations highlights a need for improved inclusivity in health data collection efforts.

**Supplementary Information:**

The online version contains supplementary material available at 10.1007/s41999-025-01231-x.

## Background

Multimorbidity, defined as the co-existence of two or more long-term health conditions, poses a significant and escalating global public health challenge [1]. Projections indicate that by 2035, approximately two-thirds of adults aged 65 and older in the UK will be living with multiple chronic conditions [2]. This trend reflects a broader global phenomenon, although estimates vary by nation. A recent systematic review found the global prevalence of multimorbidity to be 37.2%. The highest rate was seen in South America, 45.7%, followed by North America, 43.1%, Europe 39.2% and the lowest rate was seen in Asia, 35% [3].

Researchers are increasingly utilising electronic health records (EHR) as a valuable resource for investigating multimorbidity. EHR offers numerous advantages for clinical and epidemiological research, including extensive coverage of diverse population strata, easy accessibility, and the immediate availability of data that can be readily utilised for research purposes without the need for extensive data collection efforts [4, 5]. The application of EHR to multimorbidity research has gained traction, as it facilitates the linkage and integration of data on a diverse range of long-term conditions from multiple sources, such as primary care, secondary care, social care, and socioeconomic datasets. This integrative approach enhances the understanding of the aetiology, prevalence, trajectories, and cumulative risk factors associated with multimorbidity. As noted by Pearson-Stuttard, Ezzati, and Gregg [6], the rise of big data analytics in healthcare, drawing from millions of EHR entries and other datasets, offers unprecedented insights into the interrelationships among different health conditions, thereby enabling a comprehensive examination of multimorbidity within the broader health and social care systems. Moreover, EHR, particularly when combined with advanced machine learning techniques, have the potential to analyse complex variables and synthesise critical information regarding diagnosis, treatment, and outcomes across multiple conditions [7].

A recent systematic review focused specifically on patterns of multimorbidity identified across ten chronic conditions from the general patient population in 16 studies using EHR taken from primary care records [8]. This review found specific patterns and clusters of multiple long-term conditions, with mental illness featuring across all disease combinations, and called for future research to focus on underlying mechanisms behind multimorbidity and more targeted preventative interventions. Although this is a recent review, there are limitations to this work, which our study aims to address. A previous Delphi study by Dhamba-Miller (2023) [9] generated a list of 59 long-term conditions, which may yield a wider study pool than the 10 chronic conditions examined in the earlier, a focus on only primary care EHR may exclude important data. Those over 65 years are disproportionately affected by multimorbidity, and the findings from EHR may reveal more nuanced patterns and differences within this age cohort, and as a result, by adopting a singular focus on the general population, these variations may have been missed.

To the authors’ knowledge, there is no previous scoping review exploring the use of EHR to study multimorbidity in the older population. Despite the promise of EHR to facilitate large-scale multimorbidity research, there remains a lack of clarity, on: (i) how these EHRs are being utilised in studies focusing on older adults, (ii) which populations are studied from this age group, and (iii) what is known about aetiology and/or specific risk factors associated with multimorbidity? Capturing learning from previous research is essential to target preventative primary care and public health interventions, which could lessen the significant burden of multimorbidity among older generations, as well as reducing demand and costs on care providers. Specifically, this study aims to collate and summarise the existing literature on the use of electronic health records for understanding multimorbidity in individuals aged 65 and older.

## Methods

We adopted a scoping review methodology, which was based on a systematic review of the literature, aligned with the methodological framework outlined by Arksey and O’Malley (2005) [10], which consists of five stages:

Stage 1: identifying the research question,

Stage 2: identifying relevant studies,

Stage 3: study selection,

Stage 4: charting the data,

Stage 5: collating, summarizing and reporting the results.

### Identifying the research question

After reviewing the previous literature in this field, we identified several gaps that we aimed to address through the following primary research question:

How have electronic healthcare records been used to research multimorbidity in the older population?

Stemming from this, we have focused our study on three research objectives.

1. To elucidate the methodologies used in research to study older people with multimorbidity using EHR, including how multimorbidity is operationalised.
2. To explore which patient cohorts within the older multimorbidity population are being studied using EHR, which sub-groups are not represented in research, and what approaches have been taken to address this underrepresentation, including in the context of missing data.
3. To identify the key topic areas explored by the older generation multimorbidity research and highlight gaps in the current evidence.

### Identifying relevant studies

To identify relevant studies, we were assisted by a specialist medical university librarian to generate the search terms and select the most appropriate search engines. The search terms were based on previous systematic reviews using EHR and discussions between authors (NVS, LS, GS) and a professional clinical librarian (LW). The terms were then peer-reviewed by a clinical knowledge specialist using the PRESS checklist [11] (see Supplementary Materials 1 for the search terms used). Search terms included were: older people (defined as 65 years and older); routinely collected health data (that is, any data collected primarily for healthcare administration or clinical management such as health claims data, primary care and hospital electronic health records, disease registries as defined in expert-approved reporting guidance) [9]; and multimorbidity, defined as the presence of 2 or more long-term conditions from a list of 59 chronic conditions generated through national consensus work described in detail elsewhere [10] (Supplementary Materials 2).

**Fig. 1:**
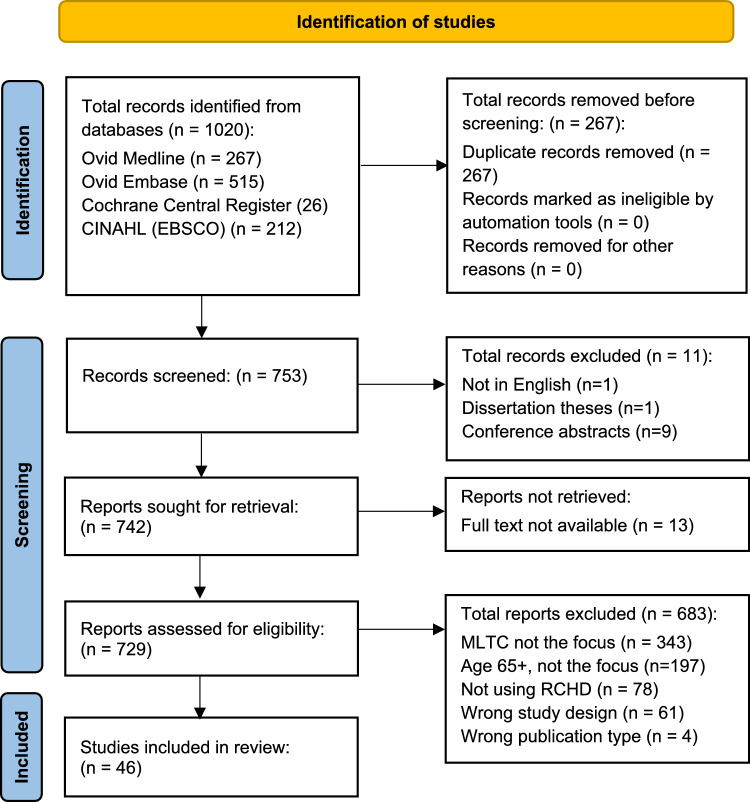
Prisma diagram showing the flow of search history and citation extraction

The searches were conducted by a medical librarian (LW) and clinical knowledge specialist (NVS) on the following databases: Medline, Embase (via Ovid), CINAHL (via EBSCO), and Cochrane Central Register of Controlled Trials, from database inception to 27/09/2024. Identified articles were extracted, de-duplicated, and added to Endnote Reference Management software. Details of the search results are provided in Fig. 1.

### Study selection: inclusion/exclusion criteria and screening

When screening for suitable studies, we used the following inclusion criteria: (1) published in the English language (with full-text available), (2) investigated populations 65 years, (3) used EHR data in their methods, and (4) the focus was on 2 or more chronic conditions from the list of 59 we previously outlined. No study design restrictions were applied, and suitable studies were not restricted to peer review articles. Reasons for exclusion were as follows: (1) the focus of the study was on populations drawn from younger than 65 years, (2) only a single chronic condition was studied, (3) the study was only available as a conference abstract, and (4) EHR data was not used.

Two researchers (NVS and LS) screened the titles and abstracts of articles identified from our initial searches by applying the inclusion and exclusion criteria. Another researcher (GS) independently screened a subset of titles and abstracts to ensure quality control. Where the research methodology was not clear or the abstract was not fully available, the full paper was read. Full copies were obtained of those papers aligned with our research questions, and these were read in full by both LS and NVS independently using the blind function on the Rayyan screening software. If the paper was excluded at this stage, a reason was provided for the other reviewer to access once unblinded. The blind function was then removed, and LS and NVS rescreened these papers. Where LS and NVS could not reach agreement on a final paper following a discussion, GS made the final decision.

### Charting the data

Articles were read in full and the data extracted by two authors (LS and NVS) into a single data extraction template on a shared Excel worksheet. The template included: paper title, year of publication, research objective, country of origin, sample sociodemographic characteristics and size, the number and types of conditions within multimorbidity, research question focus, key findings, and missingness in the data. GS audited a subsection of extracted data from each reviewer to check quality.

### Collating, summarising, and reporting the results

The extracted data was synthesised through an iterative descriptive process [12]. This process involved standard techniques used in scoping review analysis, including: (i) the use of counts to summarise the characteristics of the studies, detailing the type, number, and quality of each included paper; (ii) employing the data charting technique to order and categorise the extracted material; (iii) conducting the iterative process of ‘sifting and sorting’ [12] of the extracted material to identify patterns and common themes, which enable us to understand, interpret, and describe the data.

In practice, the analysis followed several steps. Data from the selected studies was systematically extracted into a data charting table. During the review of each paper, we identified significant characteristics and critical themes relevant to our key research objectives. Once the extraction process was completed, the research team convened to review and re-evaluate the studies, to ensure that all relevant information had been captured and our interpretations accurately reflected the extracted data.

## Results

**Objective 1:** *To elucidate the methodologies used in research to study older people with multimorbidity using EHR, including how multimorbidity is operationalised*.

### Summary of study characteristics

**Fig. 2:**
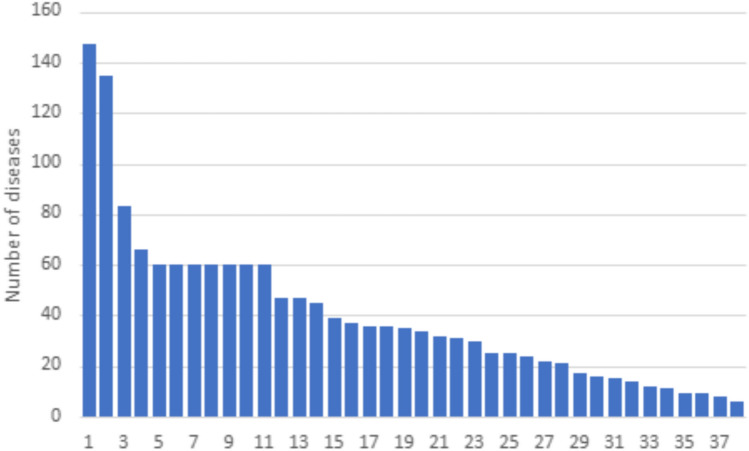
Chart showing the number of long-term conditions under the term multimorbidity within included papers* (*Paper reference number 23, 44, 48–51 did not clearly report on the number of conditions)

**Fig. 3:**
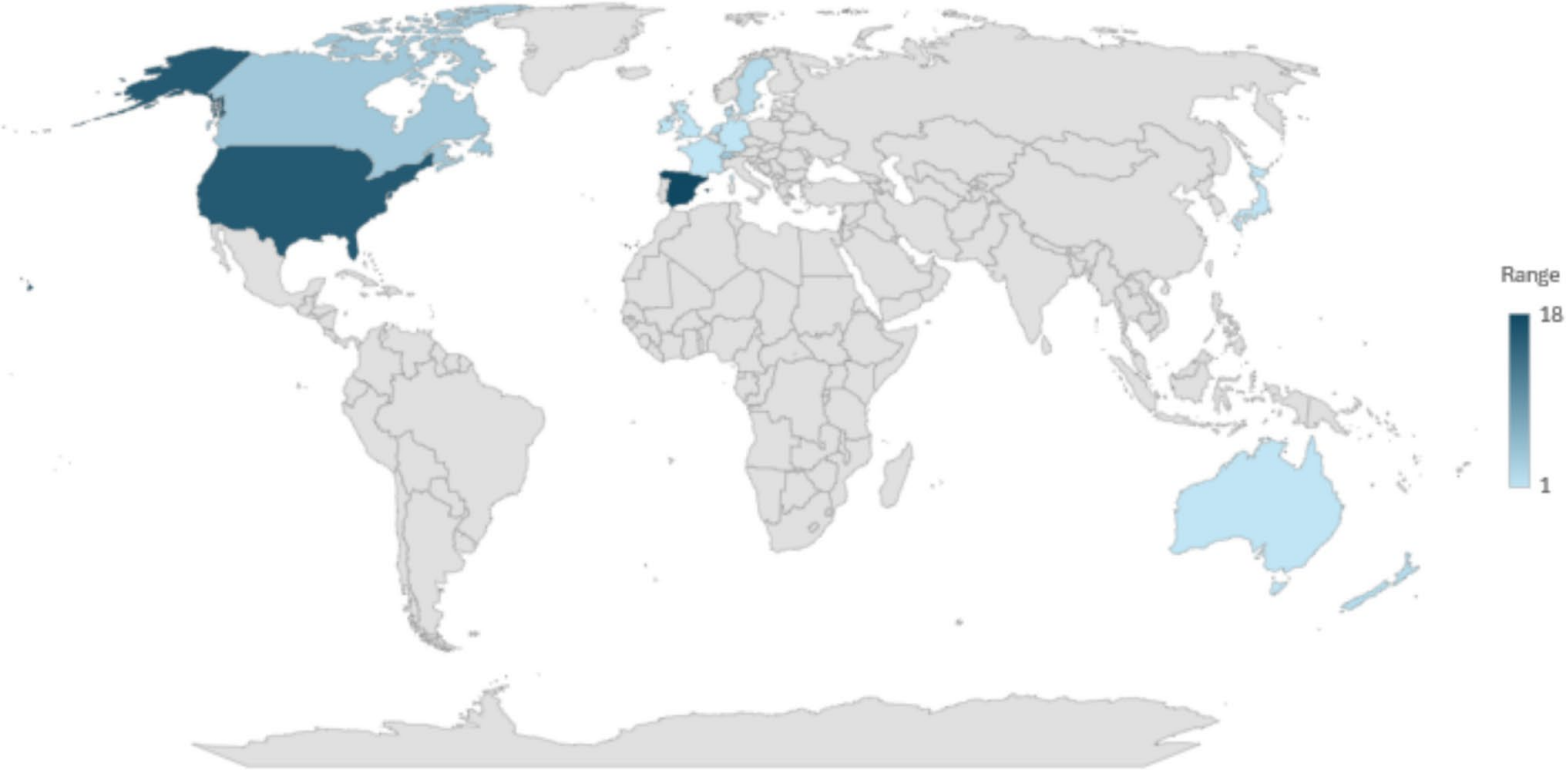
A heat map of citations by country

We identified 753 articles through searches. Of these, 46 articles met the inclusion criteria. All included studies were conducted in high-income countries (shown schematically in Fig. 3), mainly Spain, *n* = 18 = (13–31) 1], and the USA, *n* = 16 27, 32–46) [25]. There was one multinational study that included Australia, Canada, England, France, Germany, the Netherlands, New Zealand, and Spain [25], [27] Thirty-six studies included more female participants than males, all studies were predominantly on White ethnicities, and the number of conditions ranged between 6 and 147 (mean 37.40; median 31) as shown in Fig. 2. Sample sizes ranged from cohorts of 200 [25], [41] to 3,293,026,39 [41], [47]. The source of EHR varied by setting the type (i.e. primary and/or secondary, medical insurance records) and data collected (i.e. death, chronic illness prevalence, pharmacology, discharge times, admissions, expenditure). Some data was supplemented with additional measures collected directly from the patient. The characteristics of the included studies are summarised in Table 1.

**Table 1:** Study characteristics

First author, paper number, year, and (reference number)	Paper title	Age (mean/median/modal average, in years)	Ethnicity	Sample size	Sex (% female)	Number of chronic conditions	Country	Other demographic variables (not sex, age, or ethnicity)	Missingness recorded?
Abad-Díez (2014) [13]	Age and gender differences in the prevalence and patterns of multimorbidity in the older population	47.1% in the 65–74 age group	Not recorded	72,815	59.7%	32	Spain (Catalonia)	Not recorded	No
Abey-Nesbit (2023) [52]	Chronic health conditions and mortality among older adults with complex care needs in Aotearoa New Zealand	82.3	Recorded	31,704	59.9%	9	New Zealand/Aotearoa	Marital status	Yes
Akushevich (2013) [32]	Morbidity risks among older adults with pre-existing age-related diseases	Not recorded	Not recorded	2,154,598	Not recorded	21	USA	Not recorded	No
Baadoudi (2023) [53]	Are older people worse off in 2040 regarding health and resources to deal with it?	Breakdown not provided	Breakdown not provided	253,309 4,857,809 (taken from three datasets)	Breakdown not provided	9	Netherlands	Living alone, low educational level, receiving informal care, inability to meet basic needs, and insufficient self-reliance	n/a
Barrio-Cortes (2021) [14]	Chronic diseases in the geriatric population: morbidity and use of primary care services according to risk level	78.1	85.1% Spanish, 2.9% European, and 12% from the rest of the world	3292	65.8%	39	Spain	No	No
Caballero (2023) [15]	Prospective association between plasma concentrations of fatty acids and other lipids, and multimorbidity in older adults	73.6	Not recorded	1488	49.6%	60	Spain	Education level and employment	No
Carrasco-Ribelles (2023) [16]	Contribution of frailty to multimorbidity patterns and trajectories: longitudinal dynamic cohort study of aging people	69	Not recorded	1,456,052	55.8%	135	Spain (Catalonia)	Deprivation index	Yes
Carrasco-Ribelles (2022) [17]	Dynamics of multimorbidity and frailty, and their contribution to mortality, nursing home and home care nee	75	Not recorded	1,456,052	57.6%	36 60	Spain	Deprivation index, living in care home, alcohol intake, smoking	Yes
Cho (2018) [33]	Multiple chronic condition profiles and survival among oldest-old male patients with hip fracture	88	Recorded	896	0%	34	USA	Marital status, race/ethnicity, veteran/not	Yes
Dorr (2022) [34]	Prediction of future health care utilization through note-extracted psychosocial factors	73	Recorded	76,479	54%	35	USA	Chronic stress, social isolation, housing insecurity, financial insecurity, race	No
Figueroa (2021) [25]	International comparison of health spending and utilization among people with complex multimorbidity	Between 76.2 and 80.3	Not recorded	77,265	36.5–50.7%	6	Australia, Canada, England, France, Germany, the Netherlands, New Zealand, Spain, Sweden, Switzerland, and the USA	None recorded	No
Fillmore (2021) [35]	Defining multimorbidity and its impact in older United States veterans newly treated for multiple myeloma	74.8	Recorded	5076	2%	66	USA	Mean income	Yes
Fisher (2021) [36]	Effect of sociodemographic and health factors on the association between multimorbidity and acute care service use: population-based survey linked to health administrative data	65–84	Not recorded	28,361	57.4%	12	USA	Immigrant status, education, income, living arrangements, ADL, self-perceived physical and mental health	Yes
Foguet-Boreu (2015) [47]	Multimorbidity patterns in elderly primary health care patients in a South Mediterranean European Region: a cluster analysis	75.4	Not recorded	322,328	57.4%	263	Catalonia (Spain)	Not recorded	No
Gimeno-Miguel (2019) [18]	Health of Spanish centenarians: a cross-sectional study based on electronic health records	101	Not recorded	1680	79.1%	47	Spain	Deprivation	No
Green (2019) [37]	Drugs contributing to anticholinergic burden and risk of fall or fall-related injury among older adults with mild cognitive impairment, dementia and multiple chronic conditions: a retrospective cohort study	Approx. 79	Recorded	10,698	58%	186	USA	Not recorded	No
Grundy (2022) [51]	Multimorbidity as assessed by reporting of multiple causes of death in England and Wales, 2001–2017	65–95 + 80–89	Not recorded	61,676	55%	unclear	Canada	Education, household type, deprivation, marital status	No
Guisado-Clavero (2018) [19]	Multimorbidity patterns in the elderly: a prospective cohort study with cluster analysis	71.8–84.2	Not recorded	190,108	59.8%	25	Spain	Not recorded	No
Guisado-Clavero (2019) [48]	Medication patterns in older adults with multimorbidity: a cluster analysis of primary care patients	72	Not recorded	164,513	66.8%	14	Spain	None recorded	No
Hall (2020) [20]	A novel approach to developing a discordance index for older adults with chronic kidney disease	77.9	Recorded	30,932	55%	8	Spain	None recorded	No
He (2018) [38]	Prevalence of multiple chronic conditions among older adults in Florida and the United States: comparative analysis of the OneFlorida Data Trust and national inpatient sample	76.4–78	Recorded	2,635,627	Approx. 53%	25	USA	None recorded	No
Ibarra-Castillo (2018) [21]	Survival in relation to multimorbidity patterns in older adults in primary care in Barcelona, Spain (2010–2014): a longitudinal study based on electronic health records	Approx. 83	Not recorded	190,108	59.8%	147	Spain	MEDEA deprivation index	No
Ie (2017) [42]	Multimorbidity and polypharmacy in family medicine residency practices	Over 65’s, no other information given	Not recorded	1084	No recorded	24	USA	Not recorded	No
Josephson (2023) [39]	Association of comorbid-socioeconomic clusters with mortality in late-onset epilepsy derived through unsupervised machine learning	68 at onset epilepsy	Not recorded	11,307 (1048 cases, 10,259 controls)	45%	36	USA	Alcohol misuse, IMD, IQR, smoker	Yes
Jungo (2021) [29]	Utilization and spending on potentially inappropriate medications by US older adults with multiple chronic conditions using multiple medications	77.5	Not recorded	103,151	59.3%	22	Switzerland	None recorded	Yes
Jungo (2021) [50]	Baseline characteristics and comparability of older multimorbid patients with polypharmacy and general practitioners participating in a randomized controlled primary care trial	77	Recorded	323	45%	Not clear	Switzerland	Not recorded	Yes
Jungo (2021) [54]	Patient factors associated with new prescribing of potentially inappropriate medications in multimorbid US older adults using multiple medications	78	Not recorded	17,912	58.6%	17	Switzerland	None recorded	Yes
Juul‑Larsen (2019) [55]	Development of the “chronic condition measurement guide”: a new tool to measure chronic conditions in older people based on ICD-10 and ATC-codes	Cohort 1: 73 Cohort 2: 83 79	Not recorded	1: 1,083,689 2: 209,337	1: 54% 2: 53%	83	Denmark	None recorded	Yes
Kim (2018) [49]	Measuring medication adherence in older community-dwelling patients with multimorbidity	77	Not recorded	855	55%	45	Republic of Ireland	Not recorded	No
King (2015) [40]	Outpatient health care utilization in a sample of cognitively impaired veterans receiving care in VHA geriatric evaluation and management clinics	80 +	Recorded	476	5%	16	USA	Not recorded	Yes
King (2022) [41]	Accuracy of the electronic health record’s problem list in describing multimorbidity in patients with heart failure in the emergency department	78.3	Not recorded	200	52.5%	37	USA	None recorded	No
Machon (2020) [25]	Multimorbidity and functional status in older people: a cluster analysis	77.4	Not recorded	813	55.1%	15	Spain	None recorded	No
McMenamin (2024) [43]	Acute care use among patients with multiple chronic conditions receiving care from nurse practitioner practices in health professional shortage areas	75.8	Not recorded	9424	57.8%	15	USA	Education attainment, self-rated health scores	No
Ohtah (2021) [27]	Predicting factors of elderly patients’ discharge to home after rehabilitation in rural Japan: a retrospective cohort study	82.1	Not recorded	783	76.5%	Charlson Index	Japan (rural Unnan city)	Deprivation, health professional shortage area or not	Yes
Schear (2020) [44]	Multimorbidity and opioid prescribing in hospitalized older adults	75	Not recorded	238	57%	1 CIRS-G used	USA	Living with family or alone, care level	No
Simard (2024) [46]	10-Year multimorbidity trajectories in older people have limited benefit in predicting short-term health outcomes in comparison to standard multimorbidity thresholds: a population-based study	74.8	Not recorded	99,411	54.1%	31	USA	Material deprivation	No
Simard (2024) [56]	Multimorbidity prevalence and health outcome prediction: assessing the impact of lookback periods, disease count, and definition criteria in health administrative data at the population-based level	75.4	Not recorded	1,430,979	55.2%	60 Charlson and Elixhauser	Canada	Social deprivation and material deprivation	No
Stafford 48 (2021) [26]	Combined multimorbidity and polypharmacy patterns in the elderly: a cross-sectional study in primary health care	75.4	Not recorded	916,619	57.8%	47 SNAC-K	Spain (Catalonia)	Not recorded	No
Taudorf 49 (2021) [28]	Dementia increases mortality beyond effects of comorbid conditions: a national registry-based cohort study	Males: 79.6–84.1 Females: 83.1–87.1	Not recorded	1,518,917	54.0%	30 Charlson	Denmark	MEDEA Index	No
Tisminetzky (2018) [45]	Magnitude and impact of multiple chronic conditions with advancing age in older adults hospitalized with acute myocardial infarction	79	Not recorded	3863	51%	11	USA	Not recorded	No
Troncoso-Mariño (2021) [22]	Medication-related problems in older people with multimorbidity in catalonia: a real-world data study with 5 years’ follow-up	Not recorded	Not recorded	723,016	58.9%	60	Spain (Catalonia)	None recorded	No
Tsoi (2014) [30]	Medical characteristics of the oldest old: retrospective chart review of patients aged 85 + in an academic primary care centre	85–89	Not recorded	564	62.9%	Unlimited/unclear	Canada	Not recorded	No
Villen (2020) [57]	Multimorbidity patterns, polypharmacy and their association with liver and kidney abnormalities in people over 65 years of age: a longitudinal study	75.4	Not recorded	916,619 (743,827 completed follow up)	57.8%	60 SNAC-K	Spain (Catalonia)	MEDEA Index	No
Violan (2020)[23]	Five-year trajectories of multimorbidity patterns in an elderly Mediterranean population using Hidden Markov Models	75.4	Not recorded	916,619 (743,827 completed follow up)	57.7%	60 SNAC-K	Spain (Catalonia)	MEDEA Index	No
Violan (2019) [24]	Soft clustering using real-world data for the identification of multimorbidity patterns in an elderly population	75.4	Not recorded	916,619 (743,827 completed follow up)	57.7%	60 SNAC-K	Spain (Catalonia)	MEDEA Index	No
Zielinski (2015) [58]	Association between age, gender and multimorbidity level and receiving home health care: a population-based Swedish study	80 +	Not recorded	32,130	69%	Adjusted clinical groups case-mix system (ACG)	Sweden	Not recorded	No

Other study characteristics we explored, although not included in the extraction data, were follow-up periods for retrospective designs, and these were varied, making direct comparison across studies difficult. Additionally, several included studies were conducted in Catalonia, Spain, and these appeared to use the same datasets and follow-up periods. This overlap in datasets (as well as the limited geographical focus) may have skewed the representativeness of the findings, as research groups tend to adopt similar methodologies across their studies. Further, few studies included more than 1 year follow-up periods. Importantly, in terms of study quality and data validation, only two studies made any reference to reporting guidelines. Furthermore, no papers made any reference to the input or direct involvement of patient or public representatives in the research process, such as in the design, implementation, or dissemination of the research. In many countries, patient and public involvement is mandatory and regarded as an exemplar of good practice to ensure health research aligns with the ‘real-world’ priorities and expectations of service users and the wider public, especially in relation to clinical outcomes (Fig. 3).

**Objective 2:** *To explore which patient cohorts within the older multimorbidity population are being studied using EHR, which sub-groups are not represented in research, and what approaches have been taken to address this underrepresentation, including in the context of missing data*.

### Reporting of sociodemographic details amongst included articles

A number of papers reported age by mean, others by median, and some by sub-category rather than as an overall cohort. The most frequently cited ages were between 74 and 78. We found that only 11 studies (23%) included reported ethnicity 14, 21, 33–35, 37, 38, 40, 50, 52, 58 [14, 20, 25, 27, 33–35, 37, 38, 40, 46, 50]. Other minority populations typically included were Black, Hispanic, Asian, Pacific/Mauri, native Indian, and native Hawaiian. On average across all studies, over 85% of the populations were White.

**Fig. 4:**
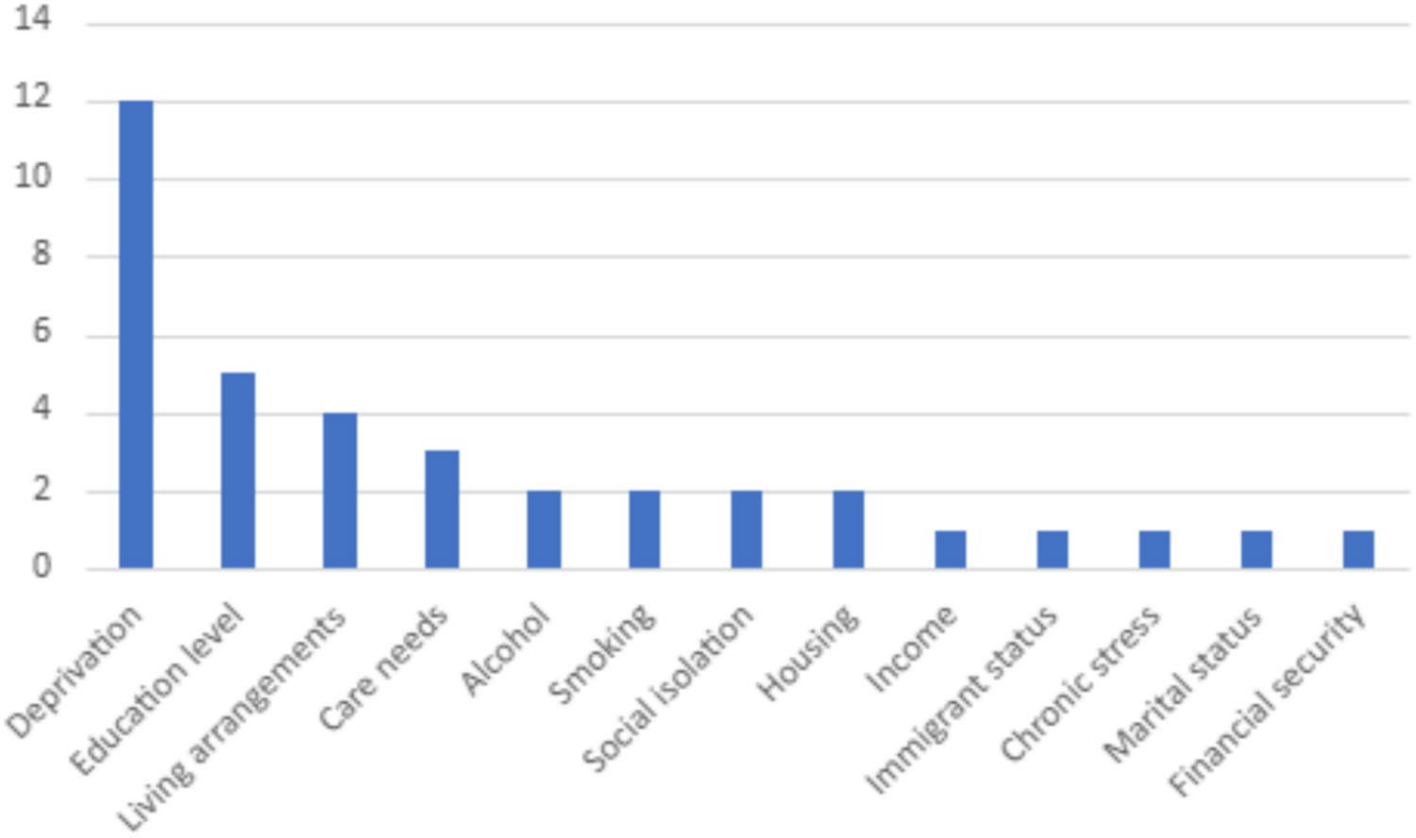
Plot showing the number of times each sociodemographic variable featured across the citations

Aside from ethnicity, few studies provided detailed baseline sociodemographic characteristics of included cohorts outside of age and sex to allow for meaningful interpretation. Twenty-two studies (47%) [15–18, 21, 23, 24, 27, 28, 33–36, 39, 43, 44, 46, 51, 52, 56, 56, 58,57,44. ] included information about other variables related to sociodemographic characteristics of included participants and is summarised in Fig. 4. Twelve out of these 20 studies that reported sociodemographic data recorded more than one demographic variable [15, 17, 27, 36, 39, 43, 44, 51, 56, 58].

Missingness of data coded in the EHRs was only reported in 13 studies (28%) [16, 17, 27, 29, 33, 35, 36, 39, 40, 50, 52, 54, 55]. In those studies that did not report missingness of data, little information was available on how this issue was addressed in the analysis.

**Objective 3:** *To identify the key topic areas explored by the older generation multimorbidity research*.

**Fig. 5:**
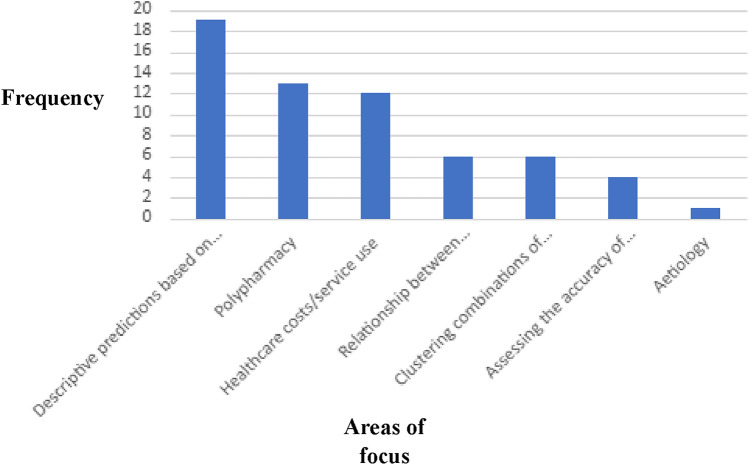
Plot showing the frequency for areas of focus across all citations

As illustrated in Fig. 5, the focus of each study can be categorised into seven themes, although some of these overlapped in certain studies. Table 2 shows the area of focus for each paper reference number.

**Table 2:** Area of focus by citation

Area of focus	Paper reference numbers and (*N*)
Healthcare costs/service use	46, 14, 17, 29, 42, 31, 51, 35, 38, 23, 41, 54 (*n* = 12)
Aetiology	15 (*n* = 1)
Descriptive predictions based on disease characteristics of patients/disease characteristics plus demographic co-variables	13, 45, 27, 29, 42, 30, 19, 32, 47, 33, 22, 52, 35, 23, 44, 5, 25 (*n* = 19)
Relationship between multimorbidity and a specific disease/health risk	16, 17, 28, 49, 41, 50 (*n* = 6)
Clustering combinations of multimorbidity	28, 18, 20, 34, 58, 26 (*n* = 6)
Polypharmacy	32, 56, 51, 43, 52, 57, 37, 39, 48, 49, 24, 55, 50 (*n* = 13)
Assessing the accuracy of EHR/chronic condition lists	21, 53, 36, 40 (*n* = 4)

Most studies (*n* = 19, 41%) discussed predicting either the likely prevalence of multimorbidity, in particular, patient groups and/or the trajectory of outcomes for different patient groups based on combinations of chronic conditions in relation to mortality or future healthcare utilisation (*n* = 12, 26%). However, among these papers, only three studies included ethnicity as a specific co-variable, with most analyses using primarily age and gender. Only one paper by Dorr et al., [34], focused specifically on sociodemographic variables, including chronic stress, social isolation, and housing and financial insecurity as risk predictors associated with chronic illness. Only six (13%) of the studies used statistical analysis to specifically identify clusters of patient groups by chronic conditions. A small number of studies focused on a specific chronic illness in relation to increased risks associated with multiple chronic conditions (*n* = 6, 13%), with dementia the most frequently examined. Polypharmacy was the second most frequent area of investigation (*n* = 13, 28%), with research focusing on medication adherence, inappropriate use of medications, de-prescribing, and healthcare utilisation/costs. Aetiology, specifically research on lipid fats, featured in only one (1%) study, indicating that EHR-driven research in relation to causation is currently underutilised. Finally, three studies (6%) considered the accuracy of EHR data, with one focusing on how chronic conditions were coded, leading to the validation of a chronic health index.

Table 3 summarises individual study findings. The data shows polypharmacy as a significant issue in the multimorbidity field, with many patients prescribed medications that are counterproductive, although it was reported that patients were willing to reduce their medication burden. The predictions explored across the studies varied, ranging from overall prevalence of multimorbidity to specific outcomes for certain chronic conditions. Several papers identified specific clusters of patients to predict risk. Collectively, these findings could inform work to target health interventions for those most at risk.

**Table 3:** Study objectives, findings, and recommendations

Authors, paper number, year	Paper title	Research objectives (taken directly from the paper)	Main findings and recommendations	Research focus theme
1. Abad-Díez (2014)	Age and gender differences in the prevalence and patterns of multimorbidity in the older population	*“To define age and gender differences in the prevalence and patterns of multimorbidity”*	67.5% of the older population have 2 or more LTCs, and this is more common in females than males, with different dominant patterns emerging (females MEC, males CM). PG pattern higher in older groups Future studies in the older population should consider stratifying by age and sex as this influences patterns and prevalence	Descriptive predictions based on disease characteristics of patients/disease characteristics plus demographic co-variables
2. Abey-Nesbit (2023)	Chronic health conditions and mortality among older adults with complex care needs in Aotearoa New Zealand	*“To determine the prevalence of chronic conditions over a three-year period and association with mortality after accounting for demographics”*	The Mauri/Pacific populations had worse outcomes for cardiovascular disease, diabetes, stroke, and cognitive degeneration and have a higher mortality rate than non-Pacific populations. This study emphasises the importance of looking at ethnic groups separately to identify whether access to assessment is equitable	Descriptive predictions based on disease characteristics of patients/disease characteristics plus demographic co-variables
3. Akushevich (2013)	Morbidity risks among older adults with pre-existing age-related diseases	*“To empirically evaluate age patterns of incidence of age-associated diseases diagnosed after the onset of earlier manifested disease and compared these patterns with those in general population.”*	For 15 disease pairs, there was 3 × the risk of subsequent disease. For 20 disease pairs, there was a lower risk of subsequent disease	Descriptive predictions based on disease characteristics of patients/disease characteristics plus demographic co-variables
4. Baadoudi (2023)	Are older people worse off in 2040 regarding health and resources to deal with it?	*“The aim of this study was to obtain insight into how the 65* + *population is distributed across the four groups of need for care and support in 2020, and the expected distribution in 2040, and to identify the developments that may affect the future distribution.”*	There are differences between MLTC prevalence projections between demographic development trajectories versus expert opinions. Experts predict double the amount of prevalence (22%) by 2040, compared to 12%. A change in psychosocial status, for example, loneliness, and changes in the presentation of multimorbidities were considered to be a driver of future prevalence amongst experts. Future research needs to focus on the psychosocial and demographic variables that may lead to an increase in complex health problems in the future, and these need to be incorporated into public health policy to redirect this trajectory	Healthcare use/costs
5. Barrio-Cortes (2021)	Chronic diseases in the geriatric population	*“The objective of this study was to describe the characteristics, morbidity, and use of services in primary care (PC) of patients with chronic diseases older than 65 years according to their risk level assigned by the adjusted morbidity groups (AMG) and to analyse the factors associated with the use of PC services.”*	The adjusted morbidity groups index, as well as older age, can be combined to predict who is most likely to use primary care services. Age is a factor which should be considered when assigning intervention level in primary care—older age carries more risk and these patients should receive a higher intervention level	Descriptive predictions based on disease characteristics of patients/disease characteristics plus demographic co-variables/healthcare use/costs
6. Caballero (2023)	Prospective Association Between Plasma Concentrations of Fatty Acids and Other Lipids, and Multimorbidity in Older Adults	*“The aim of this study was to assess the association between plasma concentrations of 9 amino acids, including branched-chain and aromatic amino acids, and multimorbidity.”*	Higher concentrations of glutamine (in women), isoleucine (possibly moderated by BMI), and valine are significantly associated with higher multimorbidity. Plasma concentrations of fatty acids should be considered as biological mechanisms which may underlie the development of multimorbidity. Lifestyle factors connected to these, which may be modifiable, need to be elucidated	Aetiology
7. Carrasco-Ribelles (2023)	Contribution of Frailty to Multimorbidity Patterns and Trajectories: Longitudinal Dynamic Cohort Study of Aging People	*“This study aimed to assess how the inclusion of frailty contributes to identifying and characterizing multimorbidity patterns in people aged 65 years or older.”*	Two distinct sets of 11 different multimorbidity patterns, based on multimorbidity and age and multimorbidity and frailty were found. Some disease appeared in both patterns but dementia and digestive motility only influenced the multimorbidity and frailty group. The age model had a better fit for death, and the frailty model had a better fit for nursing home admissions. Gender influenced patterns. Frailty needs to be considered when planning for care home services and care programmes as this can influence the type of care needed for those with multimorbidity. The different trajectories for those with multimorbidity and older versus those with multimorbidity and frailty should continue to be researched	Relationship between multimorbidity and a specific disease/health risk
8. Carrasco-Ribelles (2022)	Dynamics of multimorbidity and frailty, and their contribution to mortality, nursing home, and home care need	*“To analyse the dynamics of multimorbidity and frailty conditions as people age and calculate the associated risk of death, nursing home admission, and need for home care”*	Frailty is a major predictor of multimorbidity, with 99.8% frail patients also having multimorbidity versus 82.7% of fit individuals. This worsens with age and predicts care home usage. Future research needs to further define and describe frailty in hose over 85	Relationship between multimorbidity and a specific disease health risk Healthcare use/cost
9. Cho (2018)	Multiple chronic condition profiles and survival among oldest-old male patients with hip fracture	*“To improve understanding of survival among very elderly male patients with surgically repaired hip fractures”*	Three clusters were identified for males with hip fractures when combining multimorbidity, medication, and demographic variables: (1) low comorbidity (no prescription meds, likely to be Hispanic (2) high-longevity (longer survival), and (3) high-comorbidity (more multimorbidity, more prescriptions, and shorter lifespan). Male patients with hip fractures and multimorbidity will need more support post-discharge to increase longevity and reduce the need for increased prescribing	Clustering Relationship between multimorbidity and a specific disease/health risk
10. Dorr (2022)	Prediction of Future Health Care Utilization Through Note-extracted Psychosocial Factors	*“To examine the association of psychosocial factors with hospital utilisation amongst persons with multimorbidity.”*	Psychosocial factors can predict who was hospitalised previously, and who is more likely to have emergency department visits, mortality, and multimorbidity prevalence. Future research should consider how and when to screen for psychosocial variables as part of a cost-predictive framework	Healthcare use/cost Descriptive predictions based on disease characteristics of patients/disease characteristics plus demographic co-variables
11. Figueroa (2022)	International comparison of health spending and utilization among people with complex multimorbidity	*“The objective of this study was to explore cross-country differences in spending and utilization across different domains of care for a multimorbid persona with heart failure and diabetes.”*	Significant differences in healthcare spending and utilisation were observed across countries. The USA had the highest spending, averaging $30,877 per person on hospital care, compared to $10,956 in England. While the USA had a shorter annual hospital stay (18.9 days) than France (32.9 days) and Germany (33.4 days), it spent more on facility-based rehabilitative care. Primary care spending varied, with Australia spending $421 per person, while Spain (Aragon) spent $1,557. Specialist visits outpaced primary care visits in the USA and Canada, and across all sectors, USA expenditures were higher, likely reflecting higher prices per unit The ability to record secondary diagnoses also differed. The USA (6.3) and Germany (6.1) recorded more comorbidities per patient than Canada (3.5) and New Zealand (3.9). These variations were linked to financial incentives for diagnosis coding and the capacity of datasets. The findings suggest that countries with limited long-term care services, such as the USA, should prioritise improving access to affordable residential care to reduce reliance on costly hospital and rehabilitative facilities	Healthcare use/health cost Descriptive predictions based on disease characteristics of patients/ disease characteristics plus demographic co-variables
12. Fillmore (2021)	Defining Multimorbidity and Its Impact in Older United States Veterans Newly Treated for Multiple Myeloma	*“We aimed to define patterns of multimorbidity and their impact in older United States veterans with multiple myeloma (MM).”*	Five multimorbidity patterns were identified among older veterans: cardiovascular and metabolic diseases (30.9%), psychiatric and substance use disorders (9.7%), chronic lung disease (15.9%), multisystem impairment (13.8%), and minimal comorbidity (29.7%, reference group). Higher mortality was associated with chronic lung disease (HR 1.69), multisystem impairment (HR 2.25), and other patterns compared to minimal comorbidity. These patterns were also linked to increased emergency department visits and hospitalisations. The study highlights the importance of understanding multimorbidity patterns, rather than just the number of conditions, to improve prognosis and treatment strategies in older patients	Descriptive predictions based on disease characteristics of patients/disease characteristics plus demographic co-variables
13. Fisher (2021)	Effect of sociodemographic and health factors on the association between multimorbidity and acute care service use: population-based survey linked to health administrative data	*“This study explores how sociodemographic and health factors shape the relationship between multimorbidity and one-year acute care service use (i.e., hospital, emergency department visits) in older adults in Ontario, Canada”*	Emergency department visits and hospitalisations rose with higher multimorbidity levels. Sociodemographic factors had limited moderating effects, but patterns showed men, older adults, and those with lower incomes were more likely to use these services. Emergency visits were higher among rural residents and non-immigrants, but did not influence hospitalisations. These results underscore the importance of integrated health and social care programmes targeting multimorbidity and social determinants to optimise acute care for older adults	Descriptive predictions based on disease characteristics of patients/disease characteristics plus demographic co-variables Healthcare use/costs
14. Foguet-Boreu (2015)	Multimorbidity Patterns in Elderly Primary Health Care Patients in a South Mediterranean European Region: A Cluster Analysis	*“The purpose of this study was to identify clusters of diagnoses in elderly patients with multimorbidity, attended in primary care.”*	Cluster analysis of elderly primary care patients with multimorbidity revealed a consistent circulatory–metabolic cluster, including hypertensive diseases and metabolic disorders, as the most prevalent across sexes and age groups. A second cluster was dominated by musculoskeletal conditions, with additional conditions like varicose veins, cataracts, and dorsalgia appearing in those aged 80 and above. In women aged 65–79, a significant overlap of 54.5% was observed in the three most common diagnoses. These findings highlight key clusters for consideration in developing clinical guidelines tailored to this population	Clustering
15. Gimero-Miguel (2019)	Health of Spanish centenarians: a cross-sectional study based on electronic health records	*“With the number of centenarians increasing exponentially in Spain, a deeper knowledge of their sociodemographic, clinical, and healthcare use characteristics is important to better understand the health profile of the very elderly”*	Spanish centenarians, with an average age of 101.6 years (79.1% women), experienced multimorbidity (80%) with an average of four chronic conditions. Half were exposed to polypharmacy, averaging 4.8 medications, while only 6% had no chronic conditions, and 7% were not on medication. Multimorbidity management in this group requires a holistic geriatric approach to mitigate the risks of polypharmacy	Descriptive predictions based on disease characteristics of patients/disease characteristics plus demographic co-variables
16. Green (2019)	Drugs Contributing to Anticholinergic Burden and Risk of Fall or Fall-Related Injury among Older Adults with Mild Cognitive Impairment, Dementia and Multiple Chronic Conditions: A Retrospective Cohort Study	*“Our objective was to assess the risk of falls or fall-related injuries as a function of the overall anticholinergic score resulting from drugs with different anticholinergic ratings among people with impaired cognition.”*	Over a median follow-up of 366 days, 63% of participants used anticholinergic burden (ACB) drugs, and 18.8% experienced a fall or fall-related injury. Combining level 2 and level 3 ACB drugs significantly raised the risk of falls (HR 2.06, 95% CI 1.51–2.83), while multiple level 1 drugs also slightly increased risk (HR 1.16, 95% CI 1.03–1.32). The risk varied based on the combination of drugs within the same ACB score, with level 2 and 3 combinations posing the highest danger	Polypharmacy Descriptive predictions based on disease characteristics of patients/disease characteristics plus demographic co-variables
17. Grundy (2022)	Multimorbidity as assessed by reporting of multiple causes of death in England and Wales, 2001–2017	*“To investigate associations between recording of multiple causes of death and sociodemographic characteristics recorded at death and reported by study members at the population census prior to death. We also compare trends in number of causes of death recorded over the period 2001–2017.”*	The number of recorded causes of death increased from 2001 to 2017, peaking in those aged 85–89. It was higher among individuals with prior poor health, socioeconomic disadvantage, and those dying in hospitals, with similar counts for deaths at home or in care homes. Older age, disadvantage, and poor health are linked to more recorded conditions, highlighting multimorbidity differences and potential inconsistencies in death certification, particularly in care homes	Descriptive predictions based on disease characteristics of patients/disease characteristics plus demographic co-variables
18. Guisado-Clavero (2018)	Multimorbidity patterns in the elderly: a prospective cohort study with cluster analysis	*“The aim of this study was to identify multimorbidity patterns and their variability over a 6-year period in patients older than 65 years attended in primary health care.”*	The study identified six multimorbidity patterns, including one non-specific and five system-specific patterns: musculoskeletal, endocrine–metabolic, digestive/digestive–respiratory, neurological, and cardiovascular. The median number of chronic diseases was seven (IQR 5–10), and 42.5% of patients remained in the same pattern over time, indicating stability. These findings can aid in improving clinical management tailored to specific multimorbidity patterns	Clustering
19. Guisado-Clavero (2019)	Medication patterns in older adults with multimorbidity: a cluster analysis of primary care patients	*“The aim of our study was to describe drug prescription and medication patterns in this population”*	A study of 164,513 patients (66.8% women) found that older adults were commonly prescribed multiple medications, with 45.9% of those aged 65–79 and 61.8% of those aged 80–94 taking five or more drugs. Six medication patterns were identified, with cardiovascular and neurological drugs being the most frequently prescribed groups. Proton pump inhibitors were the most widely used, while benzodiazepines, known for potential adverse effects in older adults, were prescribed to 14.4% of men aged 65–79 and 30.2% of women aged 80–94. Understanding medication patterns in relation to multimorbidity could improve drug safety, identify adverse interactions, and aid in prioritising patient care	Polypharmacy
20. Hall (2020)	A Novel Approach to Developing a Discordance Index for Older Adults with Chronic Kidney Disease	*“Our goal is to develop a CKD-Discordance Index using electronic health records to improve recognition of discordance.”*	The study examined a cohort of older adults with a mean age of 77.9 years, 55% of whom were female and 59.3% White. Over 1 year, 32% experienced at least one hospitalisation. A CKD-Discordance Index was developed, incorporating variables like heart failure, osteoarthritis, dementia, depression, and the use of multiple prescribers. Higher index scores were associated with increased risks of hospitalisation, emergency department visits, and all-cause mortality. For example, those with an index score ≥ 0.25 had an adjusted hazard ratio of 1.81 for hospitalisation compared to those with a score of 0. The index, based on electronic health record (EHR) data, could be a valuable tool for identifying older adults with CKD-discordant conditions who are at greater risk of adverse health outcomes	Assessing the accuracy of EHR
21. He (2018)	Prevalence of Multiple Chronic Conditions Among Older Adults in Florida and the United States: Comparative Analysis of the OneFlorida Data Trust and National Inpatient Sample	*“The aim of this study was to compare the prevalence of common chronic conditions and multiple chronic conditions in older adults between Florida and the United States using data from the OneFlorida Clinical Research Consortium and the Healthcare Cost and Utilization Project (HCUP) National Inpatient Sample (NIS).”*	The most common multiple chronic condition pairs across the OneFlorida and HCUP NIS datasets included hyperlipidaemia and hypertension, hypertension and ischaemic heart disease, diabetes and hypertension, chronic kidney disease and hypertension, anaemia and hypertension, and hyperlipidaemia and ischaemic heart disease These findings confirm the high prevalence of multiple chronic conditions among older adults in Florida and across the USA. Clinical research networks like OneFlorida can serve as valuable resources for large-scale secondary data analysis	Descriptive predictions based on disease characteristics of patients/disease characteristics plus demographic co-variables
22. Ibarra-Castillo (2018)	Survival in relation to multimorbidity patterns in older adults in primary care in Barcelona, Spain (2010–2014): a longitudinal study based on electronic health records	*“The purpose of this study was to compare survival across older adults with different chronic multimorbidity patterns (CMPs).”*	Mortality rates were highest among men, individuals aged 80–94, socially disadvantaged groups, and those prescribed more drugs but with fewer conditions. Compared to the musculoskeletal pattern, men with the digestive–respiratory pattern had higher mortality risks (HR 6.16 for ages 65–79 and HR 2.62 for ages 80–94). In women, the cardiovascular pattern posed the highest risk (HR 6.34 for ages 65–79 and HR 3.05 for ages 80–94). These patterns correlated with the highest mortality rates This study highlights variations in mortality by chronic multimorbidity patterns (CMPs), age, and sex, providing valuable insights for tailoring clinical management strategies	Descriptive predictions based on disease characteristics of patients/disease characteristics plus demographic co-variables
23. Josephson (2023)	Association of comorbid–socioeconomic clusters with mortality in late onset epilepsy derived through unsupervised machine learning	*“We sought to determine if specific clusters of late onset epilepsy exist, and whether they have unique hazards of premature mortality.”*	Ten distinct phenotypic clusters were identified in late-onset epilepsy. Specific clusters had significantly higher hazards of death compared to the overall late-onset epilepsy population: ‘dementia and anxiety’ (HR 5.36), ‘brain tumour’ (HR 4.97), ‘ICH with alcohol misuse’ (HR 2.91), and ‘ischaemic stroke’ (HR 2.83). Seizure-related deaths were rare These findings demonstrate progress in providing more personalised mortality risk estimates for late-onset epilepsy patients, enabling targeted interventions for high-risk groups	Clustering
24. Jungo (2021)	Utilization and Spending on Potentially Inappropriate Medications by US Older Adults with Multiple Chronic Conditions using Multiple Medications	*“To investigate the utilization and costs of PIMs in multimorbid older adults with polypharmacy overtime.”*	In 69% of patients, at least one potentially inappropriate medication (PIM) was used. After adjusting for healthcare utilisation, chronic conditions, medication intake, and demographics, PIM use was associated with female sex (2014: OR = 1.27), older age (2014: OR = 0.92), and Hispanic ethnicity (2014: OR = 1.41). Gastrointestinal and central nervous system drugs were the most common PIMs, with over 10% of medication costs attributed to these drugs in PIM users Future initiatives should aim to optimise the prescribing of commonly used PIMs and explore sustainable deprescribing strategies. Regular PIM screenings by pharmacists or primary care physicians could improve patient safety, and interventions addressing gender differences in PIM use merit further investigation	Polypharmacy Healthcare use/cost
25. Jungo (2021)	Baseline characteristics and comparability of older multimorbid patients with polypharmacy and general practitioners participating in a randomized controlled primary care trial	*“The overall aim of this study was to determine if the GPs and patients participating in the OPTICA trial are comparable to the real-world population in Swiss primary care.”*	The GPs in the FIRE project and OPTICA trial shared similar sociodemographic and professional characteristics, such as being in their 50 s, having over 10 years of experience, and predominantly working in group practices. Patients in OPTICA were comparable to those in the FIRE database regarding age, clinical factors like blood pressure and BMI, and health service use. Notably, over 80% of multimorbid older patients expressed a willingness to stop at least one medication if advised by their doctor These findings suggest that the OPTICA trial successfully recruited a patient sample representative of the general Swiss population, making its results applicable to real-world primary care. Additionally, the high willingness for deprescribing highlights opportunities for improving medication management in multimorbid patients	Polypharmacy
26. Jungo (2021)	Patient factors associated with new prescribing of potentially inappropriate medications in us older adults with multimorbidity using multiple medications	*“The aim of this project was to investigate patient factors associated with the new outpatient prescribing of PIMs in older multimorbid adults already with polypharmacy.”*	In a study of 17,912 older adults (mean age 78) with multimorbidity and polypharmacy, 2.5% were newly prescribed a potentially inappropriate medication (PIM) within 90 days. Risk factors included male sex, frequent ambulatory visits, numerous prescribing orders, and heart failure. Patients averaged 5.1 chronic conditions and 6.1 medications. Advanced age (≥ 85 years) was associated with a reduced likelihood of PIM prescribing These findings highlight the role of clinical complexity and care coordination in PIM prescribing, suggesting a need for targeted interventions and policy changes to improve pharmacotherapy for older adults	Descriptive predictions based on disease characteristics of patients/disease characteristics plus demographic co-variables Polypharmacy
27. Juul‑Larsen (2019)	Development of the “chronic condition measurement guide”: a new tool to measure chronic conditions in older people based on ICD-10 and ATC-codes	*“The aim of the study was to develop a comprehensive open-source measurement guide of the most prevalent chronic conditions among persons aged 65* + *based on registry data of both diagnoses and prescribed drugs [the chronic condition measurement guide (CCMG)]. Furthermore, to investigate proof of concept of the measurement guide, different years of history and in- and excluding data on prescribed drugs. Finally, to investigate the measurement guide with other measurement guides designed to identify chronic conditions in persons aged 65* + *”*	We identified 83 chronic conditions based on diagnosis and prescription data. Applying the CCMG to a national cohort showed multimorbidity prevalence ranging from 10 to 69%, depending on the historical period and inclusion of prescribing data The CCMG is straightforward to implement using registry data, with 10 years of history and prescribing information recommended for optimal accuracy	Assessing the accuracy of EHR
28. Kim (2018)	Measuring medication adherence in older community-dwelling patients with multimorbidity	*“This study aimed to: (i) measure medication adherence across multiple conditions and therapeutic drug groups in older community-dwelling patients, and (ii) examine the effect of multimorbidity on adherence.”*	The median medicine possession ratio (MPR) for the cohort was 0.83, with the highest adherence observed in hypothyroidism (mean MPR = 0.88) and type 2 diabetes (mean MPR = 0.83). However, 20–40% of patients were non-adherent (MPR < 80%) across conditions. Adherence showed an inverted U-shaped relationship with the number of morbidities and drug classes, varying by morbidity burden and condition combinations Overall, 31% of older patients with multimorbidity were non-adherent to medications, highlighting the need for tailored strategies to address adherence across different conditions and treatment categories	Polypharmacy
29. King (2015)	Outpatient health care utilization in a sample of cognitively impaired veterans receiving care in VHA geriatric evaluation and management clinics	*“The present study describes the demographic characteristics, mental health diagnoses, and health care utilization of a sample of 476 VHA GEM patients with diagnosed cognitive impairment or dementia seen in clinics across Upstate New York.”*	Over 66% of veterans with diagnosed cognitive impairment or dementia had at least one additional mental health diagnosis, with many prescribed dementia and psychotropic medications, particularly antidepressants. While veterans frequently accessed mental health consultations following GEM evaluations, follow-up rates remained low These findings highlight the critical role of mental health professionals, including psychologists and psychiatrists, as collaborators in interdisciplinary geriatric care for veterans	Descriptive predictions based on disease characteristics of patients/disease characteristics plus demographic co-variables Healthcare use/cost
30. King (2022)	Accuracy of the electronic health record’s problem list in describing multimorbidity in patients with heart failure in the emergency department	*“ We assessed the accuracy of using the electronic health record (EHR) problem list to identify comorbid conditions among patients with chronic HF in the emergency department (ED).”*	The study identified the top 10 most prevalent conditions among patients with heart failure (HF) in the emergency department (ED While multimorbidity measurement using electronic health records (EHR) showed moderate-to-good accuracy, it underreported conditions like hypertension and obesity. Further research is needed to validate these methods in broader ED patient populations and hospital settings	Assessing accuracy of EHR
31 Le (2017)	Multimorbidity and Polypharmacy in Family Medicine Residency Practices	*“The aim of this research was to examine the prevalence of multimorbidity, polypharmacy, and potentially inappropriate medications (PIMs) use among older adults who visited 5 FMRPs (family medicine residency practices) more than once a year.”*	This study found that 95.6% of older adults in family medicine residency practices (FMRPs) had multimorbidity, with an average of 5.3 chronic conditions. Hypertension (87.8%), hyperlipidaemia (69.7%), and osteoarthritis (56.1%) were the most common conditions. Patients averaged 9 medications, with 86.1% meeting polypharmacy criteria (≥ 5 medications) and 33.4% prescribed at least one potentially inappropriate medication (PIM). Despite efforts, 45.4% in the polypharmacy group and 38% in the PIM group had reduced prescriptions at their last visit The findings highlight the need to improve care continuity and foster interdisciplinary collaboration to address polypharmacy and PIMs. Further research and education are essential for optimising medication management in this complex population	Polypharmacy
32. Machon (2020)	Multimorbidity and functional status in older people: a cluster analysis	*“The aim was to identify clusters of chronic diseases in robust and frail individuals and compare the sociodemographic* *and health characteristics between these clusters.”*	This study identified three health clusters among robust individuals and four among frail individuals with multimorbidity. Robust clusters ranged from better health (RC1) to higher chronic disease burden (RC2–RC3), with mobility issues prominent in RC2 and cardiovascular problems in RC3. Frail clusters also varied, with FC1 showing better health and FC4 representing individuals with severe cognitive and eye issues, as well as poorer overall health These findings provide insights into managing older patients with multimorbidity, highlighting the need for tailored approaches based on health status and cluster characteristics	Clustering
33. McMenamin (2024)	Acute Care Use Among Patients with Multiple Chronic Conditions Receiving Care from Nurse Practitioner Practices in Health Professional Shortage Areas	*“This study aims to investigate differences in hospitalization and ED utilization among Medicare beneficiaries with MCCs in these practices, comparing those who receive care in HPSAs to those in non-HPSAs.”*	Older adults with multiple chronic conditions in health professional shortage areas (HPSAs) were more likely to visit emergency departments than those in non-HPSAs, although hospitalization rates were similar This highlights disparities in emergency care use linked to workforce shortages in HPSAs. Strengthening nurse practitioner recruitment and retention in these areas could help reduce emergency visits and improve care access for patients with multiple chronic conditions	Healthcare use/cost
34. Ohtah (2021)	Predicting factors of elderly patients’ discharge to home after rehabilitation in rural Japan: a retrospective cohort study	*“The relationship between rural elderly patients’ improvement of motor and cognitive function in relation to activities of daily life (ADL) through rehabilitation and their discharge to home has not been clarified in rural communities. The purpose of this study was to clarify whether an improvement of ADL can enable elderly hospitalized patients to discharge to their homes in a rural community.”*	A high motor-FIM score after rehabilitation and a shorter rehabilitation duration were strongly associated with discharge to home, while cognitive-FIM scores were not. A motor-FIM cutoff of 60 showed good sensitivity (0.86), specificity (0.78), and a positive likelihood ratio (4.00) for predicting home discharge This study suggests that motor function improvement, rather than cognitive ability, is key to enabling elderly patients with frailty and multimorbidity to return home after rehabilitation in rural hospitals. Effective, patient-centred rehabilitation can support elderly individuals in achieving independent living at home	Descriptive predictions based on disease characteristics of patients/disease characteristics plus demographic co-variables Healthcare use/cost
35. Schear (2020)	Multimorbidity and Opioid Prescribing in Hospitalized Older Adults	*“To assess the prevalence and relationship between multimorbidity and opioid prescribing in hospitalized older medical patients with pain.”*	The average cumulative illness burden was high, with a mean CIRS-G score of 17, and 99% of patients had multimorbidity, defined as moderate-to-severe morbidity in two or more organ systems. Sixty percent were prescribed opioids at discharge, with those having higher illness burden and chronic pain significantly more likely to receive them Multimorbidity is nearly universal among older inpatients, and opioid prescriptions are common in those with higher illness burdens. Further research is needed to evaluate the risks and benefits of analgesic treatments in this population to guide clinical practice	Polypharmacy
36. Simard (2024)	10-Year Multimorbidity Trajectories in Older People Have Limited Benefit in Predicting Short-Term Health Outcomes in Comparison to Standard Multimorbidity Thresholds: A Population-Based Study	*“Our study aimed to 1) identify 10-year multimorbidity trajectories within the general population aged over 65 years, 2) evaluate the capacity of these trajectories to predict health outcomes, and 3) compare the predictive performance of multimorbidity trajectories with that of traditional cross-sectional multimorbidity indicators.”*	Eight multimorbidity trajectories were identified, covering 3% to 25% of the population, with patterns showing increasing, stable, or decreasing numbers of chronic conditions. These trajectories demonstrated similar predictive capacity for mortality and other health outcomes as traditional multimorbidity indicators, such as having three or more conditions Multimorbidity trajectories and traditional thresholds offer comparable predictive value, supporting the continued use of traditional thresholds for population surveillance and clinical practice due to their simplicity	Descriptive predictions based on disease characteristics of patients/disease characteristics plus demographic co-variables
37. Simard (2024)	Multimorbidity prevalence and health outcome prediction: assessing the impact of lookback periods, disease count, and definition criteria in health administrative data at the population-based level	*“The aim of this population-based study was to assess the impact of LP on multimorbidity prevalence and health outcomes prediction across three multimorbidity definitions, three lists of diseases used for multimorbidity assessment, and six health outcomes”*	. Predictive accuracy was highest with 10 years of data, using the L20 model and definitions for three or more conditions (MM3 +) or four or more conditions (MM4 +), demonstrating stronger predictive performance compared to less complex models Ten years of data is optimal for stable multimorbidity estimation, with MM3 + and MM4 + definitions offering the best predictive outcomes within this time frame	Assessing the accuracy of EHR
38. Stafford (2024)	Combined Multimorbidity and Polypharmacy Patterns in the Elderly: A Cross-Sectional Study in Primary Health Care	*“The aim of this study was to identify combined multimorbidity and polypharmacy patterns for the elderly population in Catalonia.”*	A seven-cluster model identified one non-specific cluster and six specific clusters linked to chronic conditions, including diabetes, neurological and musculoskeletal issues (female dominant), cardiovascular and renal diseases, and multisystem illnesses. Overrepresentation of chronic diseases and associated drug use was observed in six of the clusters These findings could inform clinical guidelines to better address patient needs and lay the groundwork for future research on the interplay between multimorbidity and polypharmacy	Polypharmacy
39. Taudorf (2021)	Dementia increases mortality beyond effects of comorbid conditions: A national registry-based cohort study	*“The aim of this study was to investigate the impact of somatic and psychiatric diseases on mortality in dementia compared with the general elderly population.”*	A higher comorbidity load was linked to increased mortality in individuals both with and without dementia. Even after accounting for psychiatric and chronic conditions, mortality rates in those with dementia remained elevated These findings indicate that dementia alone significantly contributes to excess mortality, with comorbidities further compounding the risk	Descriptive predictions based on disease characteristics of patients/disease characteristics plus demographic co-variables Relationship between MLTC and a specific disease
40. Tisminetzky (2018)	Magnitude and impact of multiple chronic conditions with advancing age in older adults hospitalized with acute myocardial infarction	*“To examine age-specific differences in the frequency and impact of cardiac and non-cardiac conditions among patients aged 65 years and older hospitalized with acute myocardial infarction (AMI).”*	Half of the patients had two or more cardiac conditions, while a third had two or more non-cardiac conditions. Patients with multiple cardiac or non-cardiac conditions faced a higher risk of severe complications or death during hospitalization compared to those with 0–1 condition The high prevalence of multimorbidity among older adults hospitalized with acute myocardial infarction (AMI) is strongly linked to worse outcomes, highlighting the need for tailored management in this vulnerable group	Healthcare use/cost Relationship with MLTC and a specific disease
41. Troncoso-Mariño (2021)	Medication-Related Problems in Older People with Multimorbidity in Catalonia: A Real-World Data Study with 5 Years’ Follow-Up	*“Aging, multimorbidity, and polypharmacy are associated with medication-related problems (MRPs). This study aimed to assess the association that multimorbidity and mortality have with MRPs in older people over time.”*	Between 2012 and 2016, the proportion of patients with at least one medication-related problem (MRP) rose from 66.9% to 75.5% (p < 0.001). Contraindicated drugs for chronic kidney disease increased from 11.1% to 18.5% and for liver disease from 3.9% to 5.3%. Potentially inappropriate medications (PIMs) rose from 62.5% to 71.1%, with fall-risk drugs being particularly common (67.5%). Patients with ten or more conditions faced higher MRP rates, including 89.6% prescribed PIMs and 34.4% contraindicated drugs for kidney disease. MRPs were linked to mortality, from duplicate therapies (HR 1.06; 95% CI 1.04–1.08) to drug interactions (HR 1.60; 95% CI 1.54–1.66) The growing prevalence of MRPs, especially in multimorbid elderly patients, presents a significant public health challenge, impacting both mortality and healthcare services. Enhanced medication management is essential to mitigate these risks	Polypharmacy
42. Tsoi (2014)	Medical characteristics of the oldest old: retrospective chart review of patients aged 85 + in an academic primary care centre	*“To describe the most common health conditions and medications used in the “oldest old”*	Patients aged 85 and older had an average of 6.4 chronic conditions and took 6.8 medications. Cardiovascular issues (79%) and bone health conditions (65%) were the most prevalent, with hypertension being the most common condition (65%). Women were more affected by bone-related conditions like osteoarthritis and osteoporosis, while coronary artery disease and type 2 diabetes were more common in men. Atorvastatin and low-dose aspirin were the most frequently prescribed medications (33% each). Men were more likely to receive lipid-lowering treatments, while women were commonly prescribed osteoporosis therapies. Lipid-lowering therapy decreased with advancing age. Multimorbidity and polypharmacy are widespread in this age group, with treatments largely targeting cardiovascular and bone health risk factors	Descriptive predictions based on disease characteristics of patients/disease characteristics plus demographic co-variables Polypharmacy
43. Villen (2020)	Multimorbidity patterns, polypharmacy and their association with liver and kidney abnormalities in people over 65 years of age: a longitudinal study	(1) To analyse medication, use according to longitudinal multimorbidity patterns (MP), and (2) determine which MP are associated with abnormal liver and kidney function	The study identified one polypharmacy profile per MP (multimorbidity patterns) and found that the most prescribed medications in each pattern matched the conditions overrepresented in that specific MP. The median of medications prescribed ranged from 3 (Cluster 1—non-specific) to 8 (Cluster 10—multisystem pattern)	Polypharmacy Relationship between MLTC and a specific disease
44. Violan (2020)	Five-year trajectories of multimorbidity patterns in an elderly Mediterranean population using Hidden Markov Models	*“This study aimed to analyse the trajectories and mortality of multimorbidity patterns in patients aged 65 to 99 years in Catalonia (Spain).”*	Twelve states, including 10 multimorbidity patterns, death, and dropouts, were identified. At baseline, the most common cluster was the non-specific pattern (42%), while the multisystem pattern was the least frequent (1.6%). Over 5 years, most participants remained in the same cluster, ranging from 92.1% in the nervous, musculoskeletal pattern to 59.2% in the cardio-circulatory and renal pattern. Mortality was highest in patterns involving cardio-circulatory diseases, such as cardio-circulatory and renal (37.1%), nervous, digestive, and circulatory (31.8%), and cardio-circulatory, mental, respiratory, and genitourinary (28.8%) This study shows that multimorbidity patterns can be tracked over time, with trajectories largely stable, but some changes observed. The hidden Markov model effectively models transitions between patterns and mortality risk, suggesting targeted health interventions for specific patterns may reduce mortality in patients with multimorbidity	Descriptive predictions based on disease characteristics of patients/disease characteristics plus demographic co-variables
45. Violan (2019)	Soft clustering using real-world data for the identification of multimorbidity patterns in an elderly population	To identify, with soft clustering methods, multimorbidity patterns in the electronic health records of a population ≥ 65 years, and to analyse such patterns in accordance with the different prevalence cutoff points applied	Multimorbidity was found in 93.1% of the population, with eight distinct disease clusters identified. These clusters varied in composition, including combinations such as nervous and digestive; respiratory, circulatory, and nervous; and mental, musculoskeletal, and genitourinary, with gender and age-related patterns observed Nuclear diseases were defined for each cluster regardless of prevalence thresholds. Fuzzy cluster analysis, which allows individuals to belong to multiple clusters simultaneously, aligns more closely with clinical practice than traditional methods commonly used in research	Clustering
46. Zielinski (2015)	Association between age, gender and multimorbidity level and receiving home health care: a population-based Swedish study	*“Our aim was to study the proportion of the population above 65 years receiving home health care according to age, gender and multimorbidity level.”*	In 2011, 7,860 individuals (28% of the studied population) received home health care. Men were 26% less likely than women to access this care (OR = 0.74, 95% CI 0.69–0.78). While 22% of recipients had low multimorbidity, higher levels of multimorbidity significantly increased the likelihood of receiving care for both genders These findings highlight disparities in home healthcare use based on gender, age, and multimorbidity. Further research is needed to explore the factors influencing care allocation and address these variations	Healthcare use/cost

Factors affecting aetiology of multimorbidity are significantly under-researched in this age cohort, especially in comparison to polypharmacy. Typically, multimorbidity patients have high levels of healthcare service usage heavily utilise health services, although significant health inequalities persist in accessing care for this cohort. However, this disparity is likely to be underestimated, due to the exclusion of patients without healthcare insurance (in those states with the healthcare model) from many of the studies using data from these sources. Notably, ethnic differences were observed in health outcomes and access, with little evidence emerging showing that health disparities are narrowing.

## Discussion

In this study, we aimed to collate, summarise, and interpret the current literature on the use of EHR to investigate multimorbidity in individuals aged over 65 years, and in doing so, we addressed three specific research objectives:

1. To elucidate the methodologies used in research to study older people with multimorbidity using EHR, including how multimorbidity is operationalised.
2. To explore which patient cohorts within the older multimorbidity population are being studied using EHR, which sub-groups are not represented in research, and what approaches have been taken to address this underrepresentation, including in the context of missing data.
3. To identify the key topic areas explored by the older generation multimorbidity research, and to highlight the gaps in the research.

In relation to the methodologies used within this area of research, variability was observed in sample sizes, reporting of sociodemographic variables, data completeness and lookback periods. This finding mirrors a recent systematic review which explored the use of EHR in multimorbidity to identify patterns, which indicates this problem is not unique [8]. Although two studies included clinicians as participants, no study reported the involvement of patients or public participants in the design, analysis, oversight and/or dissemination of research. Patient and public involvement (PPI) is increasingly recognised as essential best practice in health and social care research, to ensure research is aligned with patient needs and as a result is more likely to produce beneficial outcomes [59], [60]. The scope of long-term conditions (LTC) studied varied considerably, ranging from 6 to 147 complicating efforts to draw conclusions across studies. Further, this lack of consensus on which LTC to study hinders comparability between studies. A recent Delphi study [10] conducted in the UK developed a list of 59 long-term conditions which are now used as standard practice in UK multimorbidity research; other countries may benefit from adopting a similarly homogeneous approach to operationalising chronic conditions.

We found that many studies had retrospective study designs. This may indicate a need for more diverse longitudinal research designs to enable a more comprehensive understanding of multimorbidity over time, including evolving patterns of multiple conditions and long-term patient outcomes. Such research designs also enable assessment of the effectiveness of possible interventions in improving patient health trajectories across specific periods of time.

A significant finding of our review is that many studies highlighted concerns with the accuracy of EHR data used, particularly in the context of polypharmacy, where medications taken outside of primary care prescriptions are not captured and medication adherence is assumed. This highlights the potential risk that the findings of studies and the design of possible future interventions utilising EHR data may be inherently flawed as a result of inaccuracies in the underlying data. Three studies directly addressed the issue of EHR data accuracy. Simard [46] recommended a 10-year lookback period to enhance model stability, while Juul-Larsen [47, 55] and Hall [20, 21] focused on improving the measurement of LTC to enhance data accuracy. Although researchers are beginning to explore how to improve LTC measurement, a consistent methodological approach to strengthening data accuracy still remains in its early stages. One way to do this is to institute established guidelines for reporting research using EHR to provide a more standardised framework in this field of research. However, our finding is consistent with the results of a previous scoping review by Elstad et al. [31, 61], which found that few studies referenced these guidelines, indicating an urgent need to raise awareness of their importance in multimorbidity research going forward.

In relation to our second objective concerning study populations, an important finding to emerge from this review is that all studies were conducted in countries with established EHR systems, which are primarily a feature of modern healthcare systems in high-income states. This may have contributed to the lack of data from medium and low-income countries, potentially highlighting a significant gap in the current literature. Furthermore, as future research increasingly utilises machine learning techniques, it is critical to address the exclusion of certain populations from EHR data, as clinical interventions developed from incomplete and unrepresentative data may exacerbate existing or create new health inequalities.

Another significant contributor to gaps and missingness in data is healthcare access and utilisation trends among certain groups. Female patients were overrepresented in almost every study reviewed which likely reflects the well-established pattern of men accessing healthcare services less frequently than women, which has been attributed to women having a lower help-seeking threshold and a higher chance of survival following hospitalisation [60] 61. Further, vulnerable populations, such as immigrants, may experience fragmented care due to visiting multiple providers, leading to incomplete EHRs, whilst people from lower socioeconomic backgrounds tend to report lower healthcare utilisation [61, 62]. People from minority ethnic backgrounds also face disparities in healthcare access, as evidenced by the underreporting of ethnicity data in many studies. Additionally, in the USA, the country’s private sector insurance-based healthcare system likely exacerbates these disparities, particularly for Black Americans in terms of their health outcomes and mortality rates [63, 64]. Further, Getzen et al. [62, 65] highlight the broader consequences of missing data in EHRs, noting that this issue is under-researched and has the potential to reflect existing health inequalities. Although individuals over 65 years are more likely to access healthcare services than younger cohorts [64, 66] the review suggests that a legacy of missing data may persist among underserved populations across their lifespans. For instance, Abey-Nesbit et al. [52] found that health outcomes and the presence of chronic LTC varied by ethnicity, with Māori and Pacific patients in New Zealand facing higher mortality risks at any age. The authors stressed the importance of equitable healthcare access and the need to consider the unique contexts and characteristics of ethnic groups to assess healthcare accessibility.

Finally, in relation to our third objective, which was to identify the main topics of research in this field, gaps in the existing knowledge base are evident. Previous reviews have highlighted a lack of focus on underlying mechanisms and multimorbidity in the general population [8] and our findings reaffirm this observation of in existing research. Further, a review of the literature conducted in 2011 by Marengoni et al. on ageing and multimorbidity concluded that there is paucity of evidence on the risk factors for multimorbidity, especially in relation to genetics, biological mechanisms and environmental factors. Over 20 years later, and despite the enormity of patient data available through EHR, our review found the same research gap for those aged 65 years and over. Further, a significant gap remains in understanding the aetiology of multimorbidity in older populations. Future research should address this deficiency to enable the development of interventions targeted at the most relevant risk factors.

This review found evidence of a variety of multimorbidity clusters in older populations, based on combinations of long-term conditions (LTC) and sociodemographic factors, although there was a lack of consistency in the number of clusters found (ranging from 3–11), the amount and type of diseases included in the analyses, and other included variables and the methods used to determine these (for example, polypharmacy and social demographics). Beridze et al.’s review, which focused on patterns of multimorbidity in the general population, also found heterogeneity in the formation of clusters and number of diseases included. Although mental health and cardiovascular disease were observed in all patterns for the general population, this was not the case for clustering research for the older population. This may be because mental health is under-reported in the elderly population. For example, a recent meta-analysis of 42 studies concluded that depression prevalence is estimated to be 31% in developed countries, making it the most common psychiatric disorder for this cohort and is a significant risk marker for disability and mortality [65–69]. However, depression is underdiagnosed in 50% of cases [65, 67]. This underreporting may produce a negative bias within EHRs, potentially explaining the absence of this condition in certain clusters. Such limitations may impact the validity of EHR data when applied to older populations, highlighting the need for future research to address this issue.

In relation to patterns of multimorbidity that were identified, most studies explored variability in multimorbidity patterns, although the range of factors considered was predominantly limited to chronic condition type, age, gender, and polypharmacy. The 2015 Introduction of the Committee on the Recommended Social and Behavioural Domains and Measures for EHRs sought to address this gap by incorporating a broader range of variables that included four psychosocial factors (ethnicity, tobacco use, alcohol use, and residential address) and eight additional social economic and welfare measures (*“*education, financial resource strain, stress, depression, physical activity, social isolation, intimate partner violence, and neighbourhood median household income”) [68, 70]. Overall, however, this review found that social determinants of health (SDOH) remain underrepresented in EHR research on geriatric multimorbidity. The inherent challenges in collecting SDOH data may account for this underrepresentation in multimorbidity research. For instance, Dorr et al., [34] demonstrated the feasibility of extracting SDOH variables from patient notes to generate EHR codes, but noted that patients may be reluctant to disclose such information, and some clinicians may consider it outside of their area of practice. Future research may consider how social determinants can be better captured in EHR, so these important variables might be more easily factored into analysis.

Finally, there is a growing awareness among multimorbidity researchers that patients require holistic and personalised care delivered collaboratively by a multiple service providers and support agencies [53, 69–71]. This is necessary as the care needs of people with multimorbidity extend beyond healthcare needs, encompassing their social care and wider social support needs [71–73]. Little evidence was found of EHR-driven research addressing these wider care needs of the multimorbidity population, revealing a significant gap in the current literature. This finding highlights the needs for further research that integrates EHR data with relevant linked datasets from other fields (e.g. social care) to fully capture the complex and multifaceted care needs of this cohort.

### Strengths and limitations

To our knowledge, this scoping review is the first to specifically investigate the use of EHRs in multimorbidity research focused on older people. By mapping and collating the existing evidence in this field, this review highlighted research gaps and limitations that future studies should address. A total of 46 studies met our inclusion criteria, which is a reasonable sample size for a scoping review in terms of capturing the range and types of evidence in this field, suggesting that our search strategy was comprehensive and effective. The methodological approach employed in this review was robust. This involved a specialised librarian conducting the initial literature search, which was then peer reviewed by a colleague to ensure accuracy and thoroughness. Additionally, two independent reviewers screened all abstracts, with a third reviewer conducting a sample check to maintain consistency and minimise any possible bias during the screening process.

Our study has limitations, one of which arises from the evolving nature of terminology within this field. Multimorbidity research is still at a relatively early stage of development and as such there remains some definitional uncertainty about possible overlap or confusion with related concepts such as frailty or comorbidity, further complicating research in this field. This raises the possibility that relevant studies may not have been captured before this date if they did not explicitly use this term or its recognised synonyms. For instance, our review may not have identified some studies if they examined the co-occurrence of two or more LTC without framing the work within the multimorbidity paradigm.

We also recognise limitations in our research strategy. In particular, searches of other non-medical databases, especially more specialist databases in the fields of psychology, mental health, social sciences, and behavioural science, such as APA PsycINFO, may have yielded further relevant work relating to behavioural, psycho-social, and mental health dimensions of multimorbidity in older aged cohorts.

Although multimorbidity is more prevalent among individuals aged 65 years and older, this review found a relatively limited number of studies that specifically used EHR data to investigate multimorbidity in this demographic. Many studies were excluded due to the use of stratified age groups, with samples that included individuals with multimorbidity across a range of ages (i.e. younger, middle-aged, and older adults), making it difficult to isolate findings specific to our target population.

Another limitation relates to language and population restrictions. We included only studies published in English, which may have led to the exclusion of relevant research in other languages. Moreover, we restricted our inclusion criteria to studies that exclusively focused on cohorts aged 65 and over. Studies that included participants in this age range alongside younger cohorts were excluded, as it was challenging to disaggregate the data relevant to our target population when individuals under 65 years formed part of the overall study samples. While incorporating these studies could have increased the total number of included articles and possibly expanded the depth of our analysis, the focus of this review was EHR-driven multimorbidity research within the geriatric population. By limiting the review to studies exclusively addressing older adults, we were able to identify the way EHR is being used to specifically research this demographic. However, this exclusion criterion does mean that broader trends in multimorbidity across age ranges were not captured by this review, a subject that requires future research. Finally, all extracted studies were in high-income countries, limiting the generalisability of the findings outside these contexts.

## Conclusion

Although multimorbidity research is growing, overall, it remains a relatively under-researched field. In this context, EHRs offer a significant resource for conducting large-scale clinical epidemiological studies, particularly those focused on older populations with multimorbidity. With the voluminous quantity and breadth of EHR data now available, and when combined with the analytical and predictive potential of machine learning and artificial intelligence technologies, there exists a transformative opportunity for multimorbidity research in older populations enabling studies to be conducted on a scale previously unattainable. What is more, these technological advancements offer significant potential for the development of innovative and tailored clinical interventions to more effectively address the complex and multifactorial nature of multimorbidity.

On a cautionary note, current understanding of the psychosocial factors underlying multimorbidity is still limited. Therefore, future research using EHR to investigate multimorbidity in older populations should prioritise exploring these psychosocial determinants to inform appropriate targeted interventions. Additionally, greater reporting of completeness of data is needed to validate study findings to ensure the effective translation of research into real-world practice. Finally, it is imperative to increase the representation of underserved populations in EHR-based research. Co-producing studies with marginalised groups will ensure their views and priorities are included in studies, helping to prevent research in multimorbidity from exacerbating existing health disparities as well as contributing to improved health outcomes for all patient cohorts.

## Supplementary Information

Below is the link to the electronic supplementary material.

Supplementary file1 (DOCX 60 KB)

## References

[1] Skou ST, Mair FS, Fortin M, Guthrie B, Nunes BP, Miranda JJ et al (2022) Multimorbidity. Nat Rev Dis Primers 8(1):1–22. https://www.nature.com/articles/s41572-022-00376-4 10.1038/s41572-022-00376-4 PMC7613517 35835758

[2] Kingston A, Robinson L, Booth H, Knapp M, Jagger C, for the MODEM project (2018) Projections of multi-morbidity in the older population in England to 2035: estimates from the population ageing and care simulation (PACSim) model. Age Ageing 47(3):374–380. 10.1093/ageing/afx201 29370339 10.1093/ageing/afx201 PMC5920286

[3] Chowdhury S, Das D, Sunna T, Beyene J, Hossain A (2023) Global and regional prevalence of multimorbidity in the adult population in community settings: a systematic review and meta-analysis. Lancet eClin Med 57:101860 10.1016/j.eclinm.2023.101860 PMC9971315 36864977

[4] Benchimol EI, Smeeth L, Guttmann A, Harron K, Moher D, Petersen I et al (2015) The reporting of studies conducted using observational routinely-collected health data (RECORD) statement. PLoS Med 12(10):e1001885. 10.1371/journal.pmed.1001885 26440803 10.1371/journal.pmed.1001885 PMC4595218

[5] Nicholls SG, Langan SM, Benchimol EI (2017) Routinely collected data: the importance of high-quality diagnostic coding to research. CMAJ 189(33):E1054–E1055. https://www.cmaj.ca/content/189/33/e1054 28827435 10.1503/cmaj.170807 PMC5566604

[6] Pearson-Stuttard J, Ezzati M, Gregg EW (2019) Multimorbidity—a defining challenge for health systems. Lancet Public Health 4(12):e599-600. https://www.thelancet.com/journals/lanpub/article/PIIS2468-2667(19)30222-1/fulltext 31812234 10.1016/S2468-2667(19)30222-1

[7] Witham MD, Cooper R, Missier P, Robinson SM, Sapey E, Sayer AA (2023) Researching multimorbidity in hospital: can we deliver on the promise of health informatics? Eur Geriatr Med 14(4):765–768. 10.1007/s41999-023-00753-6 37227692 10.1007/s41999-023-00753-6 PMC10447588

[8] Beridze G, Abbadi A, Ars J, Remelli F, Vetrano DL, Trevisan C, Pérez LM, López-Rodríguez JA, Calderón-Larrañaga A (2024) Patterns of multimorbidity in primary care electronic health records: a systematic review. J Multimorb Comorb 14:26335565231223350. 10.1177/26335565231223350. (**PMID: 38298757; PMCID: PMC10829499**) 38298757 10.1177/26335565231223350 PMC10829499

[9] Dambha-Miller H, Farmer A, Nirantharakumar K et al (2023) Artificial Intelligence for multiple long-term conditions (AIM): a consensus statement from the NIHR AIM consortia [version 1; not peer reviewed]. NIHR Open Res 3:21. 10.3310/nihropenres.1115210.1

[10] Arksey H, O’Malley L (2005) Scoping studies: towards a methodological framework. Int J Soc Res Methodol 8(1):19–32. 10.1080/1364557032000119616

[11] McGowan J, Sampson M, Salzwedel DM, Cogo E, Foerster V, Lefebvre C (2016) PRESS peer review of electronic search strategies: 2015 guideline statement. J Clin Epidemiol 75:40–46. 10.1016/j.jclinepi.2016.01.021. (**Epub 2016 Mar 19 PMID: 27005575**) 27005575 10.1016/j.jclinepi.2016.01.021

[12] Dambha-Miller H, Cheema S, Saunders N, Simpson G (2022) Multiple long-term conditions (MLTC) and the environment: a scoping review. Int J Environ Res Public Health 19(18):11492. 10.3390/ijerph191811492 36141763 10.3390/ijerph191811492 PMC9517156

[13] Abad-Díez JM, Calderón-Larrañaga A, Poncel-Falcó A, Poblador-Plou B, Calderón-Meza JM, Sicras-Mainar A, Clerencia-Sierra M, Prados-Torres A (2014) Age and gender differences in the prevalence and patterns of multimorbidity in the older population. BMC Geriatr 14:75. 10.1186/1471-2318-14-75. (**PMID: 24934411; PMCID: PMC4070347**) 24934411 10.1186/1471-2318-14-75 PMC4070347

[14] Barrio-Cortes J, Castaño-Reguillo A, Beca-Martínez MT et al (2021) Chronic diseases in the geriatric population: morbidity and use of primary care services according to risk level. BMC Geriatr 21:278. 10.1186/s12877-021-02217-7 33902470 10.1186/s12877-021-02217-7 PMC8074273

[15] Caballero FF, Lana A, Struijk EA, Arias-Fernández L, Yévenes-Briones H, Cárdenas-Valladolid J, Salinero-Fort MÁ, Banegas JR, Rodríguez-Artalejo F, Lopez-Garcia E (2023) Prospective association between plasma concentrations of fatty acids and other lipids, and multimorbidity in older adults. J Gerontol A Biol Sci Med Sci 78(10):1763–1770. 10.1093/gerona/glad122. (**PMID: 37156635**) 37156635 10.1093/gerona/glad122

[16] Carrasco-Ribelles LA, Cabrera-Bean M, Danés-Castells M, Zabaleta-Del-Olmo E, Roso-Llorach A, Violán C (2023) Contribution of frailty to multimorbidity patterns and trajectories: longitudinal dynamic cohort study of aging people. JMIR Public Health Surveill 9:e45848. 10.2196/45848. (**PMID: 37368462; PMCID: PMC10365626**) 37368462 10.2196/45848 PMC10365626

[17] Carrasco-Ribelles LA, Roso-Llorach A, Cabrera-Bean M, Costa-Garrido A, Zabaleta-Del-Olmo E, Toran-Monserrat P, Orfila Pernas F, Violán C (2022) Dynamics of multimorbidity and frailty, and their contribution to mortality, nursing home and home care need: a primary care cohort of 1456052 ageing people. EClinicalMedicine 11(52):101610. 10.1016/j.eclinm.2022.101610. (**PMID: 36034409; PMCID: PMC9399153**) 10.1016/j.eclinm.2022.101610 PMC9399153 36034409

[18] Gimeno-Miguel A, Clerencia-Sierra M, Ioakeim I, Poblador-Plou B, Aza-Pascual-Salcedo M, González-Rubio F, Rodríguez Herrero R, Prados-Torres A (2019) Health of Spanish centenarians: a cross-sectional study based on electronic health records. BMC Geriatr 19(1):226. 10.1186/s12877-019-1235-7. (**PMID: 31426764; PMCID: PMC6701024**) 31426764 10.1186/s12877-019-1235-7 PMC6701024

[19] Guisado-Clavero M, Roso-Llorach A, López-Jimenez T, Pons-Vigués M, Foguet-Boreu Q, Muñoz MA, Violán C (2018) Multimorbidity patterns in the elderly: a prospective cohort study with cluster analysis. BMC Geriatr 18(1):16. 10.1186/s12877-018-0705-7. (**PMID: 29338690; PMCID: PMC5771078**) 29338690 10.1186/s12877-018-0705-7 PMC5771078

[20] Hall RK, Zhou H, Reynolds K, Harrison TN, Bowling CB (2020) A novel approach to developing a discordance index for older adults with chronic kidney disease. J Gerontol A Biol Sci Med Sci 75(3):522–528. 10.1093/gerona/glz248. (**PMID: 31644788; PMCID: PMC7768728**) 31644788 10.1093/gerona/glz248 PMC7768728

[21] Ibarra-Castillo C, Guisado-Clavero M, Violan-Fors C, Pons-Vigués M, López-Jiménez T, Roso-Llorach A (2018) Collaborators. Survival in relation to multimorbidity patterns in older adults in primary care in Barcelona, Spain (2010–2014): a longitudinal study based on electronic health records. J Epidemiol Commun Health 72(3):185–192. 10.1136/jech-2017-209984. (**Epub 2018 Jan 12 PMID: 29330165**) 10.1136/jech-2017-209984 29330165

[22] Troncoso-Mariño A, Roso-Llorach A, López-Jiménez T, Villen N, Amado-Guirado E, Fernández-Bertolin S, Carrasco-Ribelles LA, Borras JM, Violán C (2021) Medication-related problems in older people with multimorbidity in Catalonia: a real-world data study with 5 years’ follow-up. J Clin Med 10(4):709. 10.3390/jcm10040709. (**PMID: 33670201; PMCID: PMC7916946**) 33670201 10.3390/jcm10040709 PMC7916946

[23] Violán C, Fernández-Bertolín S, Guisado-Clavero M et al (2020) Five-year trajectories of multimorbidity patterns in an elderly Mediterranean population using hidden Markov models. Sci Rep 10:16879. 10.1038/s41598-020-73231-9 33037233 10.1038/s41598-020-73231-9 PMC7547668

[24] Violán C, Foguet-Boreu Q, Fernández-Bertolín S, Guisado-Clavero M, Cabrera-Bean M, Formiga F, Valderas JM, Roso-Llorach A (2019) Soft clustering using real-world data for the identification of multimorbidity patterns in an elderly population: cross-sectional study in a Mediterranean population. BMJ Open 9(8):e029594. 10.1136/bmjopen-2019-029594. (**PMID: 31471439; PMCID: PMC6719769**) 31471439 10.1136/bmjopen-2019-029594 PMC6719769

[25] Figueroa JF, Papanicolas I, Riley K, Abiona O, Arvin M, Atsma F, Bernal-Delgado E, Bowden N, Blankart CR, Deeny S, Estupiñán-Romero F, Gauld R, Haywood P, Janlov N, Knight H, Lorenzoni L, Marino A, Or Z, Penneau A, Shatrov K, van de Galien O, van Gool K, Wodchis W, Jha AK (2021) International comparison of health spending and utilization among people with complex multimorbidity. Health Serv Res 56(Suppl 3):1317–1334. 10.1111/1475-6773.13708. (**Epub 2021 Aug 5. PMID: 34350586; PMCID: PMC8579210**) 34350586 10.1111/1475-6773.13708 PMC8579210

[26] Stafford G, Villén N, Roso-Llorach A, Troncoso-Mariño A, Monteagudo M, Violán C (2021) Combined multimorbidity and polypharmacy patterns in the elderly: a cross-sectional study in primary health care. Int J Environ Res Public Health 18(17):9216. 10.3390/ijerph18179216. (**PMID: 34501805; PMCID: PMC8430667**) 34501805 10.3390/ijerph18179216 PMC8430667

[27] Ohta R, Maeki N, Maniwa S, Miyakoshi K (2021) Predicting factors of elderly patients’ discharge to home after rehabilitation in rural Japan: a retrospective cohort study. Rural Remote Health 21(1):6406. 10.2605/RRH6406. (**Epub 2021 Jan 7. PMID: 33405939**) 33405939 10.22605/RRH6406

[28] Taudorf L, Nørgaard A, Brodaty H, Laursen TM, Waldemar G (2021) Dementia increases mortality beyond effects of comorbid conditions: a national registry-based cohort study. Eur J Neurol 28(7):2174–2184. 10.1111/ene.14875. (**Epub 2021 May 14. PMID: 33894084; PMCID: PMC8251545**) 33894084 10.1111/ene.14875 PMC8251545

[29] Jungo KT, Streit S, Lauffenburger JC (2021) Utilization and spending on potentially inappropriate medications by US older adults with multiple chronic conditions using multiple medications. Arch Gerontol Geriatr 93:104326. 10.1016/j.archger.2020.104326. (**Epub 2020 Dec 20 PMID: 33516154**) 33516154 10.1016/j.archger.2020.104326

[30] Tsoi CS, Chow JY, Choi KS, Li HW, Nie JX, Tracy CS, Wang L, Upshur RE (2014) Medical characteristics of the oldest old: retrospective chart review of patients aged 85+ in an academic primary care centre. BMC Res Notes 7:340. 10.1186/1756-0500-7-340. (**PMID: 24897943; PMCID: PMC4061508**) 24897943 10.1186/1756-0500-7-340 PMC4061508

[31] Zenebe Y, Akele B, W/Selassie M et al (2021) Prevalence and determinants of depression among old age: a systematic review and meta-analysis. Ann Gen Psychiatry 20:55. 10.1186/s12991-021-00375-x 34922595 10.1186/s12991-021-00375-x PMC8684627

[32] Akushevich I, Kravchenko J, Ukraintseva S, Arbeev K, Kulminski A, Yashin AI (2013) Morbidity risks among older adults with pre-existing age-related diseases. Exp Gerontol 48(12):1395–1401. 10.1016/j.exger.2013.09.005. (**Epub 2013 Sep 21. PMID: 24064264; PMCID: PMC3895485**) 24064264 10.1016/j.exger.2013.09.005 PMC3895485

[33] Cho J, Stock EM, Liao IC, Zeber JE, Ahmedani BK, Basu R, Quinn CC, Copeland LA (2018) Multiple chronic condition profiles and survival among oldest-old male patients with hip fracture. Arch Gerontol Geriatr 74:184–190. 10.1016/j.archger.2017.10.014. (**Epub 2017 Oct 28. PMID: 29126081; PMCID: PMC5737015**) 29126081 10.1016/j.archger.2017.10.014 PMC5737015

[34] Dorr DA, Quiñones AR, King T, Wei MY, White K, Bejan CA (2022) Prediction of future health care utilization through note-extracted psychosocial factors. Med Care 60(8):570–578. 10.1097/MLR.0000000000001742. (**Epub 2022 Jun 4. PMID: 35658116; PMCID: PMC9262845**) 35658116 10.1097/MLR.0000000000001742 PMC9262845

[35] Fillmore NR, DuMontier C, Yildirim C, La J, Epstein MM, Cheng D, Cirstea D, Yellapragada S, Abel GA, Gaziano JM, Do N, Brophy M, Kim DH, Munshi NC, Driver JA (2021) Defining multimorbidity and its impact in older united states veterans newly treated for multiple myeloma. J Natl Cancer Inst 113(8):1084–1093. 10.1093/jnci/djab007. (**PMID: 33523236; PMCID: PMC8328982**) 33523236 10.1093/jnci/djab007 PMC8328982

[36] Fisher KA, Griffith LE, Gruneir A, Upshur R, Perez R, Favotto L, Nguyen F, Markle-Reid M, Ploeg J (2021) Effect of socio-demographic and health factors on the association between multimorbidity and acute care service use: population-based survey linked to health administrative data. BMC Health Serv Res 21(1):62. 10.1186/s12913-020-06032-5. (**PMID: 33435978; PMCID: PMC7805153**) 33435978 10.1186/s12913-020-06032-5 PMC7805153

[37] Green AR, Reifler LM, Bayliss EA, Weffald LA, Boyd CM (2019) Drugs contributing to anticholinergic burden and risk of fall or fall-related injury among older adults with mild cognitive impairment, dementia and multiple chronic conditions: a retrospective cohort study. Drugs Aging 36(3):289–297. 10.1007/s40266-018-00630-z. (**PMID: 30652263; PMCID: PMC6386184**) 30652263 10.1007/s40266-018-00630-z PMC6386184

[38] He Z, Bian J, Carretta HJ, Lee J, Hogan WR, Shenkman E, Charness N (2018) Prevalence of multiple chronic conditions among older adults in Florida and the United States: comparative analysis of the OneFlorida Data Trust and national inpatient sample. J Med Internet Res 20(4):e137. 10.2196/jmir.8961. (**PMID: 29650502; PMCID: PMC5920146**) 29650502 10.2196/jmir.8961 PMC5920146

[39] Josephson CB, Gonzalez-Izquierdo A, Engbers JDT, Denaxas S, Delgado-Garcia G, Sajobi TT, Wang M, Keezer MR, Wiebe S (2023) Association of comorbid-socioeconomic clusters with mortality in late onset epilepsy derived through unsupervised machine learning. Seizure 111:58–67. 10.1016/j.seizure.2023.07.016. (**Epub 2023 Jul 29 PMID: 37536152**) 37536152 10.1016/j.seizure.2023.07.016

[40] King PR, Vair CL, Wade M, Gass J, Wray LO, Kusche A, Saludades C, Chang J (2015) Outpatient health care utilization in a sample of cognitively impaired veterans receiving care in VHA geriatric evaluation and management clinics. Psychol Serv 12(1):66–72. 10.1037/ser0000015. (**Epub 2014 Nov 24. PMID: 25419916**) 25419916 10.1037/ser0000015

[41] King BL, Meyer ML, Chari SV, Hurka-Richardson K, Bohrmann T, Chang PP, Rodgers JE, Busby-Whitehead J, Casey MF (2022) Accuracy of the electronic health record’s problem list in describing multimorbidity in patients with heart failure in the emergency department. PLoS ONE 17(12):e0279033. 10.1371/journal.pone.0279033. (**PMID: 36512600; PMCID: PMC9747000**) 36512600 10.1371/journal.pone.0279033 PMC9747000

[42] Ie K, Felton M, Springer S, Wilson SA, Albert SM (2017) Multimorbidity and polypharmacy in family medicine residency practices. J Pharm Technol 33(6):219–224. 10.1177/8755122517725327. (**Epub 2017 Aug 11. PMCID: PMC5998471**)

[43] McMenamin A, Turi E, Dixon J, Liu J, Martsolf G, Poghosyan L (2024) Acute care use among patients with multiple chronic conditions receiving care from nurse practitioner practices in health professional shortage areas. Nurs Res 73(5):E212–E220. 10.1097/NNR.0000000000000758. (**Epub 2024 Jun 26. PMID: 38989998; PMCID: PMC11344658**) 38989998 10.1097/NNR.0000000000000758 PMC11344658

[44] Schear S, Patel K, Deng LX, Miaskowski C, Maravilla I, Garrigues SK, Thompson N, Auerbach AD, Ritchie CS (2020) Multimorbidity and opioid prescribing in hospitalized older adults. J Palliat Med 23(4):475–482. 10.1089/jpm.2019.0260. (**Epub 2019 Nov 5. PMID: 31689152; PMCID: PMC7643761**) 31689152 10.1089/jpm.2019.0260 PMC7643761

[45] Tisminetzky M, Nguyen HL, Gurwitz JH, McManus D, Gore J, Singh S, Yarzebski J, Goldberg RJ (2018) Magnitude and impact of multiple chronic conditions with advancing age in older adults hospitalized with acute myocardial infarction. Int J Cardiol 272:341–345. 10.1016/j.ijcard.2018.08.062. (**Epub 2018 Aug 22. PMID: 30172472; PMCID: PMC6173997**) 30172472 10.1016/j.ijcard.2018.08.062 PMC6173997

[46] Simard M, Rahme E, Dubé M, Boiteau V, Talbot D, Mésidor M, Chiu YM, Sirois C (2024) 10-Year multimorbidity trajectories in older people have limited benefit in predicting short-term health outcomes in comparison to standard multimorbidity thresholds: a population-based study. Clin Epidemiol 16:345–355. 10.2147/CLEP.S456004. (**PMID: 38798914; PMCID: PMC11128253**) 38798914 10.2147/CLEP.S456004 PMC11128253

[47] Foguet-Boreu Q, Violán C, Rodriguez-Blanco T, Roso-Llorach A, Pons-Vigués M, Pujol-Ribera E, Cossio Gil Y, Valderas JM (2015) Multimorbidity patterns in elderly primary health care patients in a south Mediterranean European region: a cluster analysis. PLoS ONE 10(11):e0141155. 10.1371/journal.pone.0141155. (**PMID: 26524599; PMCID: PMC4629893**) 26524599 10.1371/journal.pone.0141155 PMC4629893

[48] Guisado-Clavero M, Violán C, López-Jimenez T, Roso-Llorach A, Pons-Vigués M, Muñoz MA, Foguet-Boreu Q (2019) Medication patterns in older adults with multimorbidity: a cluster analysis of primary care patients. BMC Fam Pract 20(1):82. 10.1186/s12875-019-0969-9. (**PMID: 31195985; PMCID: PMC6567459**) 31195985 10.1186/s12875-019-0969-9 PMC6567459

[49] Kim S, Bennett K, Wallace E, Fahey T, Cahir C. Measuring medication adherence in older community-dwelling patients with multimorbidity. Eur J Clin Pharmacol. 2018;74(3):357–64. 10.1007/s00228-017-2388-y. Epub 2017 Dec 3. PMID: 29199370.58. Committee on the Recommended Social and Behavioral Domains and Measures for Electronic Health Records; Board on Population Health and Public Health Practice; Institute of Medicine. Capturing Social and Behavioral Domains and Measures in Electronic Health Records: Phase 2. Washington (DC): National Academies Press (US); 2015 Jan 8. Committee on the recommended social and behavioral domains and measures for electronic health records. Available from https://www.ncbi.nlm.nih.gov/books/NBK269337/. 10.1007/s00228-017-2388-y 29199370

[50] Jungo KT, Meier R, Valeri F, Schwab N, Schneider C, Reeve E, Spruit M, Schwenkglenks M, Rodondi N, Streit S (2021) Baseline characteristics and comparability of older multimorbid patients with polypharmacy and general practitioners participating in a randomized controlled primary care trial. BMC Fam Pract 22(1):123. 10.1186/s12875-021-01488-8. (**PMID: 34157981; PMCID: PMC8220761**) 34157981 10.1186/s12875-021-01488-8 PMC8220761

[51] Grundy EM, Stuchbury R (2022) Multimorbidity as assessed by reporting of multiple causes of death: variations by period, sociodemographic characteristics and place of death among older decedents in England and Wales, 2001–2017. J Epidemiol Commun Health. 76(8):699–706. 10.1136/jech-2021-217846. (**Epub ahead of print. PMID: 35654580; PMCID: PMC9279827**) 10.1136/jech-2021-217846 PMC9279827 35654580

[52] Abey-Nesbit R, Jamieson HA, Bergler HU, Kerse N, Pickering JW, Teh R (2023) Chronic health conditions and mortality among older adults with complex care needs in Aotearoa New Zealand. BMC Geriatr 23(1):318. 10.1186/s12877-023-03961-8. (**PMID: 37217895; PMCID: PMC10201728**) 37217895 10.1186/s12877-023-03961-8 PMC10201728

[53] Baadoudi F, Picavet, S, Hildrink H, Hendrix, R, Rijken M, Bruin S., (2023) Are older people worse off in 2040 regarding health and resources to deal with it? - Future developments in complex health problems and in the availability of resources to manage health problems in the Netherlands Front. Public Health, 16 June 2023 Sec. Aging and Public Health Volume 11-2023 10.3389/fpubh.2023.942526 10.3389/fpubh.2023.942526 PMC10311544 37397729

[54] Jungo KT, Streit S, Lauffenburger JC (2021) Patient factors associated with new prescribing of potentially inappropriate medications in multimorbid US older adults using multiple medications. BMC Geriatr 21(1):163. 10.1186/s12877-021-02089-x. (**PMID: 33676398; PMCID: PMC7937195**) 33676398 10.1186/s12877-021-02089-x PMC7937195

[55] Juul-Larsen HG, Christensen LD, Andersen O, Bandholm T, Kaae S, Petersen J (2019) Development of the “chronic condition measurement guide”: a new tool to measure chronic conditions in older people based on ICD-10 and ATC-codes. Eur Geriatr Med 10(3):431–444. 10.1007/s41999-019-00188-y. (**Epub 2019 Apr 10 PMID: 34652799**) 34652799 10.1007/s41999-019-00188-y

[56] Simard M, Rahme E, Dubé M, Boiteau V, Talbot D, Sirois C (2024) Multimorbidity prevalence and health outcome prediction: assessing the impact of lookback periods, disease count, and definition criteria in health administrative data at the population-based level. BMC Med Res Methodol 24(1):113. 10.1186/s12874-024-02243-0. (**PMID: 38755529; PMCID: PMC11097445**) 38755529 10.1186/s12874-024-02243-0 PMC11097445

[57] Villén N, Guisado-Clavero M, Fernández-Bertolín S, Troncoso-Mariño A, Foguet-Boreu Q, Amado E, Pons-Vigués M, Roso-Llorach A, Violán C (2020) Multimorbidity patterns, polypharmacy and their association with liver and kidney abnormalities in people over 65 years of age: a longitudinal study. BMC Geriatr 20(1):206. 10.1186/s12877-020-01580-1. (**Erratum in: BMC Geriatr. 2022 May 6;22(1):399. 10.1186/s12877-021-02567-2. PMID: 32532213; PMCID: PMC7291454**) 32532213 10.1186/s12877-020-01580-1 PMC7291454

[58] Zielinski A, Halling A (2015) Association between age, gender and multimorbidity level and receiving home health care: a population-based Swedish study. BMC Res Notes 8:714. 10.1186/s13104-015-1699-2. (**PMID: 26602364; PMCID: PMC4658801**) 26602364 10.1186/s13104-015-1699-2 PMC4658801

[59] Elstad M, Ahmed S, Røislien J, Douiri A (2023) Evaluation of the reported data linkage process and associated quality issues for linked routinely collected healthcare data in multimorbidity research: a systematic methodology review. BMJ Open 13(5):e069212. 10.1136/bmjopen-2022-069212. (**Published 2023 May 8**) 37156590 10.1136/bmjopen-2022-069212 PMC10174005

[60] Gianfrancesco MA, Tamang S, Yazdany J, Schmajuk G (2018) Potential biases in machine learning algorithms using electronic health record data. JAMA Intern Med 178(11):1544–1547. 10.1001/jamainternmed.2018.3763. (**PMID: 30128552; PMCID: PMC6347576**) 30128552 10.1001/jamainternmed.2018.3763 PMC6347576

[61] Odonkor CA, Sholas MG, Verduzco-Gutierrez M, Zafonte RD, Silver JK (2020) African American patient disparities in COVID-19 out-comes: a call to action for physiatrists to provide rehabilitation care to Black survivors. Am J Phys Med Rehabil. 10.1097/PHM.000000000000156 32804715 10.1097/PHM.0000000000001568 PMC7526402

[62] Simons K, Bradfield O, Spittal MJ et al (2023) Age and gender patterns in health service utilisation: age-period-cohort modelling of linked health service usage records. BMC Health Serv Res 23:480. 10.1186/s12913-023-09456-x 37173743 10.1186/s12913-023-09456-x PMC10176675

[63] Getzen E, Ungar L, Mowery D, Jiang X, Long Q (2023) Mining for equitable health: assessing the impact of missing data in electronic health records. J Biomed Inf 139:104269. 10.1016/j.jbi.2022.104269. (**Epub 2023 Jan 5. PMID: 36621750; PMCID: PMC10391553**) 10.1016/j.jbi.2022.104269 PMC10391553 36621750

[64] Gray-Burrows KA, Willis TA, Foy R, Rathfelder M, Bland P, Chin A, Hodgson S, Ibegbuna G, Prestwich G, Samuel K, Wood L, Yaqoob F, McEachan RRC (2018) Role of patient and public involvement in implementation research: a consensus study. BMJ Qual Saf 27(10):858–864. 10.1136/bmjqs-2017-006954. (**Epub 2018 Apr 17. PMID: 29666310; PMCID: PMC6166593**) 29666310 10.1136/bmjqs-2017-006954 PMC6166593

[65] James SL, Abate D, Abate KH, Abay SM, Abbafati C, Abbasi N et al (2018) Global, regional, and national incidence, prevalence, and years lived with disability for 354 diseases and injuries for 195 countries and territories, 1990–2017: a systematic analysis for the Global Burden of Disease Study 2017. Lancet 392(10159):1789–1858 30496104 10.1016/S0140-6736(18)32279-7 PMC6227754

[66] Blazer DG, Hybels CF, Pieper CF (2001) The association of depression and mortality in elderly persons: a case for multiple, independent pathways. J Gerontol A Biol Sci Med Sci 56(8):M505–M509 11487603 10.1093/gerona/56.8.m505

[67] Dambha-Miller H, Simpson G, Hobson L et al (2021) Integrating primary care and social services for older adults with multimorbidity: a qualitative study. Br J Gen Pract 71(711):e753–e761. 10.3399/BJGP.2020.1100. (**Published 2021 Sep 30**) 34019480 10.3399/BJGP.2020.1100 PMC8436775

[68] Höhn A, Gampe J, Lindahl-Jacobsen R, Christensen K, Oksuyzan A (2020) Do men avoid seeking medical advice A register-based analysis of gender-specific changes in primary healthcare use after first hospitalisation at ages 60+ in Denmark. J Epidemiol Commun Health 74(7):573–579. 10.1136/jech-2019-213435. (**Epub 2020 Apr 17. PMID: 32303595; PMCID: PMC7337231**) 10.1136/jech-2019-213435 PMC7337231 32303595

[69] Simpson G, Stuart B, Hijryana M et al (2023) Eliciting and prioritising determinants of improved care in multimorbidity: a modified online Delphi study. J Multimorb Comorb. 10.1177/26335565231194552 37692105 10.1177/26335565231194552 PMC10483969

[70] Simpson G, Stokes J, Farmer A, Dambha-Miller H (2023) Social care need in multimorbidity. J R Soc Med 116(4):124–127. 10.1177/01410768231168382 37078268 10.1177/01410768231168382 PMC10164274

[71] Simpson G, Morrison L, Santer M, Hijryana M, Farmer A, Dambha-Miller H (2024) Perceptions and experiences of living with and providing care for multimorbidity: a qualitative interview study. J Multimorb Comorb. 10.1177/26335565241240820 38529048 10.1177/26335565241240820 PMC10962039

[72] Machón M, Mateo-Abad M, Clerencia-Sierra M, Güell C, Poblador-Pou B, Vrotsou K, Gimeno-Miguel A, Prados-Torres A, Vergara I. Multimorbidity and functional status in older people: a cluster analysis. Eur Geriatr Med. 2020 Apr;11(2):321-332. 10.1007/s41999-020-00291-5. Epub 2020 Feb 17. PMID: 32297200 10.1007/s41999-020-00291-5 32297200

[73] Committee on the Recommended Social and Behavioral Domains and Measures for Electronic Health Records; Board on Population Health and Public Health Practice; Institute of Medicine. Capturing Social and Behavioral Domains and Measures in Electronic Health Records: Phase 2. Washington (DC): National Academies Press (US); 2015 Jan 8. Committee on the recommended social and behavioral domains and measures for electronic health records. Available from: https://www.ncbi.nlm.nih.gov/books/NBK269337/. 25590118

