# Supplementary material for: Using electronic health records to understand multimorbidity in older people: a scoping review
Source: Eur Geriatr Med. 2025 Jul 3;16(4):1163–206. doi: 10.1007/s41999-025-01231-x (PMC12378290; doi:10.1007/s41999-025-01231-x)
Supplement: Supplementary file 1 — Supplementary file1 (DOCX 60 KB) [file 41999_2025_1231_MOESM1_ESM.docx]

**Supplementary Information 1: Summary of search terms**

| **MeSH and Free Text Search Terms** | **Databases** | **Filters/Refined by** | **Number of Sources identified** |
| --- | --- | --- | --- |
| **Ovid MEDLINE**(R) ALL <1946 to September 24, 2024>  1 Multimorbidity/ 3478  2 (multimorbid* or multi-morbid* or "multiple long term condition*" or "multiple long-term condition*" or MLTC).ti,ab. 11616  3 1 or 2 11985  4 routinely collected health data/ 307  5 ("routin* collected data" or "routin* collected health* data" or "Routin*-Collected Health* Data" or "routin*-collected data").ti,ab. 2450  6 (health adj2 "admin* data").ti,ab. 1331  7 "registr* data".ti,ab. 12813  8 "wearable device data".ti,ab. 84  9 Electronic Health Records/ 29652  10 ("electronic health record*" or "electronic medical record*").ti,ab. 61125  11 4 or 5 or 6 or 7 or 8 or 9 or 10 88903  12 exp Aged/ 3572620  13 ("65 years or older" or "70 years or older" or "75 years or older" or "80 years or older").ti,ab. 10790  14 ("older than 65" or "older than 70" or "older than 75" or "older than 80").ti,ab. 10697  15 (Aged or elder* or frail or geriatric*).ti,ab. 1090123  16 ("old* people" or "old* person").ti,ab. 45940  17 (senior adj3 citi*).ti,ab. 1803  18 12 or 13 or 14 or 15 or 16 or 17 4210774  19 3 and 11 and 18 **267** | Ovid MEDLINE | Searches from database inception.  Language: restricted to the English language. | **267** |
| Query Limiters/Expanders Last Run Via Results  S19 S3 AND S11 AND S18. Search modes – Proximity Interface - EBSCOhost Research Databases  Search Screen - Advanced Search.  Database - CINAHL Ultimate - **212**  S18 S12 OR S13 OR S14 OR S15 OR S16 OR S17. Search modes – Proximity Interface - EBSCOhost Research Databases.Search Screen - Advanced Search. Database - CINAHL Ultimate 1,177,056  S17 TI (senior N3 citi*) OR AB (senior N3 citi*) Search modes – Proximity Interface - EBSCOhost Research Databases. Search Screen - Advanced Search. Database - CINAHL Ultimate 886  S16 TI ( ("old* people" or "old* person") ) OR AB ( ("old* people" or "old* person") ) Search modes – Proximity Interface - EBSCOhost Research Databases. Search Screen - Advanced Search. Database - CINAHL Ultimate 30,911  S15 TI ( (Aged or elder* or frail or geriatric*) ) OR AB ( (Aged or elder* or frail or geriatric*) ) Search modes - Proximity Interface - EBSCOhost Research Databases. Search Screen - Advanced Search. Database - CINAHL Ultimate 372,164  S14 TI ( ("older than 65" or "older than 70" or "older than 75" or "older than 80") ) OR AB ( ("older than 65" or "older than 70" or "older than 75" or "older than 80") ) Search modes – Proximity Interface - EBSCOhost Research Databases. Search Screen - Advanced Search. Database - CINAHL Ultimate. 5,346  S13 TI ( ("65 years or older" or "70 years or older" or "75 years or older" or "80 years or older") ) OR AB ( ("65 years or older" or "70 years or older" or "75 years or older" or "80 years or older") ) Search modes – Proximity Interface - EBSCOhost Research Databases. Search Screen - Advanced Search. Database - CINAHL Ultimate. 9,001  S12 (MH "Aged+") Search modes – Proximity Interface - EBSCOhost Research Databases. Search Screen - Advanced Search. Database - CINAHL Ultimate 966,978  S11 S4 OR S5 OR S6 OR S7 OR S8 OR S9 OR S10 Search modes – Proximity Interface - EBSCOhost Research Databases. Search Screen - Advanced Search. Database - CINAHL Ultimate 55,283  S10 TI ( ("electronic health record*" or "electronic medical record*") ) OR AB ( ("electronic health record*" or "electronic medical record*") ) Search modes – Proximity Interface - EBSCOhost Research Databases. Search Screen - Advanced Search. Database - CINAHL Ultimate 27,955  S9 (MH "Electronic Health Records+") Search modes – Proximity Interface - EBSCOhost Research Databases. Search Screen - Advanced Search. Database - CINAHL Ultimate 32,001  S8 TI "wearable device data" OR AB "wearable device data" Search modes - Proximity Interface - EBSCOhost Research Databases  Search Screen - Advanced Search Database - CINAHL Ultimate 16  S7 TI "registr* data" OR AB "registr* data" Search modes – Proximity Interface - EBSCOhost Research Databases. Search Screen - Advanced Search. Database - CINAHL Ultimate 4,310  S6 TI (health N2 "admin* data") OR AB (health N2 "admin* data") Search modes - Proximity Interface - EBSCOhost Research Databases Search Screen - Advanced Search. Database - CINAHL Ultimate 734  S5 TI ( ("routin* collected data" or "routin* collected health* data" or "Routin*-Collected Health* Data" or "routin*-collected data") ) OR AB ( ("routin* collected data" or "routin* collected health* data" or "Routin*-Collected Health* Data" or "routin*-collected data") ) Search modes - Proximity Interface - EBSCOhost Research Databases Search Screen - Advanced Search. Database - CINAHL Ultimate 937  S4 (MH "Routinely Collected Health Data") Search modes – Proximity Interface - EBSCOhost Research Databases. Search Screen - Advanced Search. Database - CINAHL Ultimate 36  S3 S1 OR S2 Search modes – Proximity Interface - EBSCOhost Research Databases. Search Screen - Advanced Search. Database - CINAHL Ultimate 19,997  S2 TI ( (multimorbid* or multi-morbid* or "multiple long term condition*" or "multiple long-term condition*" or MLTC) ) OR AB ( (multimorbid* or multi-morbid* or "multiple long term condition*" or "multiple long-term condition*" or MLTC) ) Search modes – Proximity Interface - EBSCOhost Research Databases  Search Screen - Advanced Search. Database - CINAHL Ultimate 5,279  S1 (MH "Morbidity") Search modes – Proximity Interface - EBSCOhost Research Databases. Search Screen - Advanced Search. Database - CINAHL Ultimate 14,949 | CINAHL (EBSCO) | Searches from database inception.  Language: restricted to the English language. | **212** |
| **Embase** <1974 to 2024 Week 38>    1 multiple chronic conditions/ 10342  2 (multimorbid* or multi-morbid* or "multiple long term condition*" or "multiple long-term condition*" or MLTC).ti,ab. 15441  3 1 or 2 18805  4 routinely collected health data/ 403  5 ("routin* collected data" or "routin* collected health* data" or "Routin*-Collected Health* Data" or "routin*-collected data").ti,ab. 3282  6 (health adj2 "admin* data").ti,ab. 1927  7 "registr* data".ti,ab. 20857  8 "wearable device data".ti,ab. 106  9 exp electronic health record/ 51060  10 ("electronic health record*" or "electronic medical record*").ti,ab. 110890  11 4 or 5 or 6 or 7 or 8 or 9 or 10 151335  12 exp aged/ 3942021  13 ("65 years or older" or "70 years or older" or "75 years or older" or "80 years or older").ti,ab. 14415  14 ("older than 65" or "older than 70" or "older than 75" or "older than 80").ti,ab. 17646  15 (Aged or elder* or frail or geriatric*).ti,ab. 1509851  16 ("old* people" or "old* person").ti,ab. 57009  17 (senior adj3 citi*).ti,ab. 2244  18 12 or 13 or 14 or 15 or 16 or 17 4807480  19 3 and 11 and 18  **515** | EMBASE | Searches from database inception.  Language: restricted to the English language. | **515** |
| ID Search Hits  #1 MeSH descriptor: [Multimorbidity] this term only 162  #2 (multimorbid* or multi-morbid* or multiple long term NEXT condition* or multiple long-term NEXT condition* or MLTC):ti,ab 955  #3 #1 or #2 968  #4 MeSH descriptor: [Routinely Collected Health Data] this term only 13  #5 (routin* collected NEXT data or routin* collected health* NEXT data or Routine-Collected Health* NEXT Data or routine-collected NEXT data):ti,ab 578  #6 (health NEAR/2 admin* NEXT data):ti,ab 63  #7 (registr* NEXT data):ti,ab 835  #8 "wearable device data":ti,ab 12  #9 MeSH descriptor: [Electronic Health Records] this term only 876  #10 (electronic health NEXT record* or electronic medical NEXT record*):ti,ab 5883  #11 #4 or #5 or #6 or #7 or #8 or #9 or #10 7486  #12 MeSH descriptor: [Aged] explode all trees 278605  #13 ("65 years or older" or "70 years or older" or "75 years or older" or "80 years or older"):ti,ab 1860  #14 ("older than 65" or "older than 70" or "older than 75" or "older than 80"):ti,ab 1265  #15 (Aged or elder* or frail or geriatric*):ti,ab 219820  #16 (old* NEXT people or old* NEXT person):ti,ab 6950  #17 (senior NEAR/3 citi*):ti,ab 179  #18 #12 or #13 or #14 or #15 or #16 or #17 463829  #19 #3 and #11 and #18 **26** | Cochrane Library | Searches from database inception.  Language: restricted to the English language. | **26** |

**Supplementary Information 2**

**List of 59 included chronic health care conditions (10)**

| **Condition number** | **Chronic condition** |
| --- | --- |
|  | MLTC |
|  | Stroke |
|  | Coronary heart disease |
|  | Heart failure |
|  | Peripheral arterial disease |
|  | Heart valve disorder |
|  | Arrthymia |
|  | Venous thromboembolism |
|  | Aneurysm |
|  | Hypertension (Treated and untreated) |
|  | Diabetes |
|  | Addisons disease |
|  | Cystic fibrosis |
|  | Thyroid disorders |
|  | COPD |
|  | Asthma |
|  | Bronchiectasis |
|  | Parkinsons |
|  | Epilepsy |
|  | MS |
|  | Paralysis |
|  | Transient ischaemic attack |
|  | Peripheral neuropathy |
|  | Chronic primary pain |
|  | Solid organ cancer |
|  | Haematological cancer |
|  | Metastatic cancer |
|  | Melanoma |
|  | Benign cerebral tumours causing disability |
|  | Dementia |
|  | Schizophrenia |
|  | Depression |
|  | Anxiety |
|  | Bipolar disorder |
|  | Drug or alcohol misuse |
|  | Eating disorder |
|  | Autism |
|  | Post-traumatic stress disorder |
|  | Connective tissue disorder |
|  | Osteoarthritis |
|  | Osteoporosis |
|  | Gout |
|  | Long-term musculoskeletal problem due to injury |
|  | Chronic liver disease |
|  | Inflammatory bowel |
|  | Chronic pancreatic disease |
|  | Peptic ulcer |
|  | Chronic kidney disease---stage ¾ |
|  | End-stage kidney disease |
|  | Endometriosis |
|  | Chronic urinary tract infection |
|  | Anaemia (including pernicious anaemia and sickle cell anaemia) |
|  | Vsual impairment that cannot be corrected |
|  | Hearing impairment that cannot be corrected |
|  | Meniere's disease |
|  | HIV/AIDS |
|  | Chronic Lyme Disease |
|  | TB |
|  | Post-acute COVID-19 disease |
